# RCO-3 and COL-26 form an external-to-internal module that regulates the dual-affinity glucose transport system in *Neurospora crassa*

**DOI:** 10.1186/s13068-021-01877-2

**Published:** 2021-01-28

**Authors:** Jinyang Li, Qian Liu, Jingen Li, Liangcai Lin, Xiaolin Li, Yongli Zhang, Chaoguang Tian

**Affiliations:** 1 Key Laboratory of Systems Microbial Biotechnology, Tianjin Institute of Industrial Biotechnology, Chinese Academy of Sciences, Tianjin, 300308 China; 2 University of Chinese Academy of Sciences, Beijing, 100049 China; 3 State Key Laboratory of Agrobiotechnology and MOA Key Laboratory of Soil Microbiology, College of Biological Sciences, China Agricultural University, Beijing, 100193 China; 4 National Technology Innovation Center of Synthetic Biology, Tianjin, 300308 China

**Keywords:** *Neurospora crassa*, Phosphoproteome, Glucose transport, Transcription factor, Gene regulation, RNA-seq

## Abstract

**Background:**

Low- and high-affinity glucose transport system is a conserved strategy of microorganism to cope with environmental glucose fluctuation for their growth and competitiveness. In *Neurospora crassa*, the dual-affinity glucose transport system consists of a low-affinity glucose transporter GLT-1 and two high-affinity glucose transporters HGT-1/HGT-2, which play diverse roles in glucose transport, carbon metabolism, and cellulase expression regulation. However, the regulation of this dual-transporter system in response to environmental glucose fluctuation is not yet clear.

**Results:**

In this study, we report that a regulation module consisting of a downstream transcription factor COL-26 and an upstream non-transporting glucose sensor RCO-3 regulates the dual-affinity glucose transport system in *N. crassa*. COL-26 directly binds to the promoter regions of *glt-1*, *hgt-1*, and *hgt-2*, whereas RCO-3 is an upstream factor of the module whose deletion mutant resembles the Δ*col-26* mutant phenotypically. Transcriptional profiling analysis revealed that Δ*col-26* and Δ*rco-3* mutants had similar transcriptional profiles, and both mutants had impaired response to a glucose gradient. We also showed that the AMP-activated protein kinase (AMPK) complex is involved in regulation of the glucose transporters. AMPK is required for repression of *glt-1* expression in starvation conditions by inhibiting the activity of RCO-3.

**Conclusions:**

RCO-3 and COL-26 form an external-to-internal module that regulates the glucose dual-affinity transport system. Transcription factor COL-26 was identified as the key regulator. AMPK was also involved in the regulation of the dual-transporter system. Our findings provide novel insight into the molecular basis of glucose uptake and signaling in filamentous fungi, which may aid in the rational design of fungal strains for industrial purposes.

## Background

Glucose is the preferred carbon source for most microorganisms as well as a signaling molecule that regulates physiological and pathological processes [1]. The sensing and uptake of glucose triggers a cellular regulatory network that influences multiple biological processes including sugar transporter expression, carbon catabolism, and biomass accumulation [2]. In cellulolytic filamentous fungi, the detection of glucose triggers the repression of genes encoding lignocellulose-degrading enzymes, a mechanism known as carbon catabolite repression (CCR), which is mediated by the transcription factor CreA/CRE1 [3, 4]. Although CreA/CRE1-mediated transcriptional repression has been extensively studied [3, 5–9], sensing of extracellular glucose concentrations and subsequent transport are barely characterized in filamentous fungi.

In *Saccharomyces cerevisiae*, two transporter-like glucose sensors, Rgt2p and Snf3p, mediate a glucose signaling pathway [10, 11]. Upon glucose detection, Rgt2p/Snf3p-associated casein kinase I (Yck1p/Yck2p) phosphorylates Mth1p and Std1p [12], leading to their degradation by SCF^Grr1^-mediated proteolysis [13], which triggers the release of Rgt1p from hexose transporter (HXT) promoter regions and derepresses the *HXT* genes [14]. Extracellular glucose concentrations are also detected by a G-protein coupled receptor, Gpr1p. Binding of glucose to Gpr1p activates the downstream heterotrimeric G*α* protein Gpa2p [15]. The Gpr1p/Gpa2p pathway works in parallel with Ras to activate adenylate cyclase Cyr1p, which increases cAMP levels, thereby activating protein kinase A (PKA). The cAMP/PKA pathway is important for spore germination, hyphal growth, cell wall homeostasis, conidiation, and secondary metabolite production [16–19]. PKA catalyzes phosphorylation of Rgt1p and regulates its function [20, 21], suggesting some crosstalk between these two sensing pathways.

The signaling pathway required to adapt to nutrient limitation and to use alternative carbon sources centers on the kinase Snf1p, a component of the *S. cerevisiae* SNF1 complex [22, 23]. The SNF1 complex is homologous to AMP-activated protein kinase (AMPK) in higher eukaryotes, which acts as a regulator of cellular energy homeostasis [24]. Like AMPK, SNF1 is a heterotrimer consisting of a catalytic *α*-subunit (Snf1p), a regulatory γ-subunit (Snf4p), and one of three *β*-subunits (Sip1p, Sip2p, or Gal83p) [25, 26]. In low glucose conditions, Snf1p is activated by the phosphorylation of residue Thr210 by one of three upstream kinases (Sak1p, Tos3p, or Elm1p) [27–29]. In response to high glucose concentrations, Snf1p is inactivated by dephosphorylation of Thr210 by phosphatase Glc7p [30]. ADP association with Snf4p protects Thr210 of Snf1p from dephosphorylation [31]. Active Snf1p phosphorylates the inhibitor Mig1p, the homolog of CreA/CRE1 in filamentous fungi, to relieve CCR through its translocation to the cytoplasm [32], promoting the use of alternative carbon sources such as ethanol and acetate [33]. Snf1p also phosphorylates Cyr1p and negatively regulates PKA-dependent transcription [34], whereas PKA phosphorylates the Snf1-activating kinase Sak1p [35] and the *β*-subunit Sip1p [36], suggesting a crosstalk between the Snf1p and PKA pathways. Snf1p/Mig1p, Rgt2p/Snf3p, and cAMP/PKA pathways, as well as their crosstalk, enable yeast cells to sense a wide range of glucose concentrations and subsequently express corresponding transporters such as low-affinity glucose transporters Hxt1p and Hxt3p or high-affinity glucose transporters Hxt6p and Hxt7p [37, 38].

The low-affinity and high-affinity glucose uptake systems in filamentous fungi were described a long time ago [39], but the corresponding genes were characterized at the molecular level much later [39–41]. In *Aspergillus niger*, MstA, MstF, MstG, and MstH were determined to be high-affinity glucose transporters, whereas MstC is a low-affinity glucose transporter [42–44]. In *A. nidulans*, MstE is a low-affinity glucose transporter, whereas HxtA, MstA (HxtD), and MstC (HxtB) were described as high-affinity glucose transporters [45–48], and HxtC and HxtE are glucose transporters with unknown affinity [47]. MstC (HxtB) is also involved in glucose signaling and metabolism in *A. nidulans* [49]. In *N. crassa*, GLT1 was characterized as a low-affinity glucose transporter, and HGT-1 and HGT-2 are two high-affinity glucose transporters [50, 51]. GLT-1 and HGT-1/-2 were identified as the major components of the dual-affinity glucose transport system of *N. crassa* with HGT-1/-2 also involved in glucose signaling and CCR [51]. In *Colletotrichum graminicola*, CgHXT1 and CgHXT3 are glucose transporters with high-affinity, whereas the *A. nidulans* MstE and *N. crassa* GLT1 ortholog CgHXT5 is a low-affinity glucose transporter [52]. Other characterized high-affinity glucose transporters include GTT1 from *Trichoderma harzianum* [53]; UfHXT1p from *Uromyces fabae* [54]; the *N. crassa* RCO-3 ortholog AmMst1 from *Amanita muscaria* [55, 56]; RiMST2, RiMST5, and RiMST6 from *Rhizophagus irregularis* [57, 58]; and GpMST1 from *Geosiphon pyriformis* [59]. Glucose transporters with unknown affinity include Stp1 and TrHxt1 from *Trichoderma reesei* [60, 61], and MSF1 from *Colletotrichum lindemuthianum*, whose deletion mutant resembles to some extent the Δ*rco-3* mutant of *N. crassa* in growth and conidiation [62].

However, in contrast to the comprehensive analysis of hexose transport in *S. cerevisiae*, much less is known about glucose sensing and the subsequent transcriptional regulation of glucose transporter-encoding genes in filamentous fungi. In *N*. *crassa*, the *S. cerevisiae* Gpr1p ortholog GPR-4 functions as a carbon source receptor. Ligand binding to GPR-4 activates the downstream G*α* protein GNA-1, leading to an increase in cAMP level produced by the activated adenylate cyclase CR-1 [63]. Similarly, in *A*. *nidulans*, the G-protein-coupled receptor GprH and G*α* subunit GanB have been shown to be involved in glucose sensing [64, 65]. During early conidial germination events, GanB activates cAMP synthesis and subsequent PKA activity in the presence of glucose [64]. In *Ustilago maydis*, the *S. cerevisiae* Rgt2p/Snf3p ortholog Hxt1 was characterized as a high-affinity glucose transporter and sensor involved in glucose signaling [66]. Similarly in *N. crassa*, the yeast Rgt2p/Snf3p ortholog RCO-3 was shown to be involved in glucose sensing [67]. Mutation of this non-transporting glucose sensor leads to complete dysfunction of the low-affinity glucose transport system and partial impairment of the high-affinity system [67]. However, orthologs of *S. cerevisiae* Mth1p, Sth1p, and Rgt1p have not been found in filamentous fungi, suggesting the presence of a different signaling pathway from the *S. cerevisiae* Rgt2p/Snf3p pathway.

In this study, we identified a module including transcription factor COL-26, the ortholog of the Zn(II)_2_Cys_6_ transcription factor AmyR in *Aspergillus* [68, 69], and RCO-3, a non-transporting glucose sensor, that regulate the dual-affinity glucose transport system in the model fungus *N. crassa*. COL-26 binds to the promoter regions of *glt-1* and *hgt-1/-2* to promote their expression, and RCO-3 is also essential for *glt-1* expression in both glucose-rich and starvation conditions, and regulates the pathway, possibly by indirectly affecting the expression of COL-26. Transcriptomic analysis showed that the response of Δ*col-26* and Δ*rco-3* mutants to a glucose gradient was greatly impaired. In addition, AMPK is also involved in the pathway by inhibiting the activity of RCO-3 in starvation conditions. Since the dual-affinity glucose transport system is widely conserved among fungal species, knowledge about its regulation will provide a foundation for further investigation into the molecular basis of nutrient transport and signaling as well as plant cell wall degradation in fungi.

## Results

### Identification of COL-26 as a key transcription factor to promote *glt-1* expression in response to external glucose

**Fig. 1:**
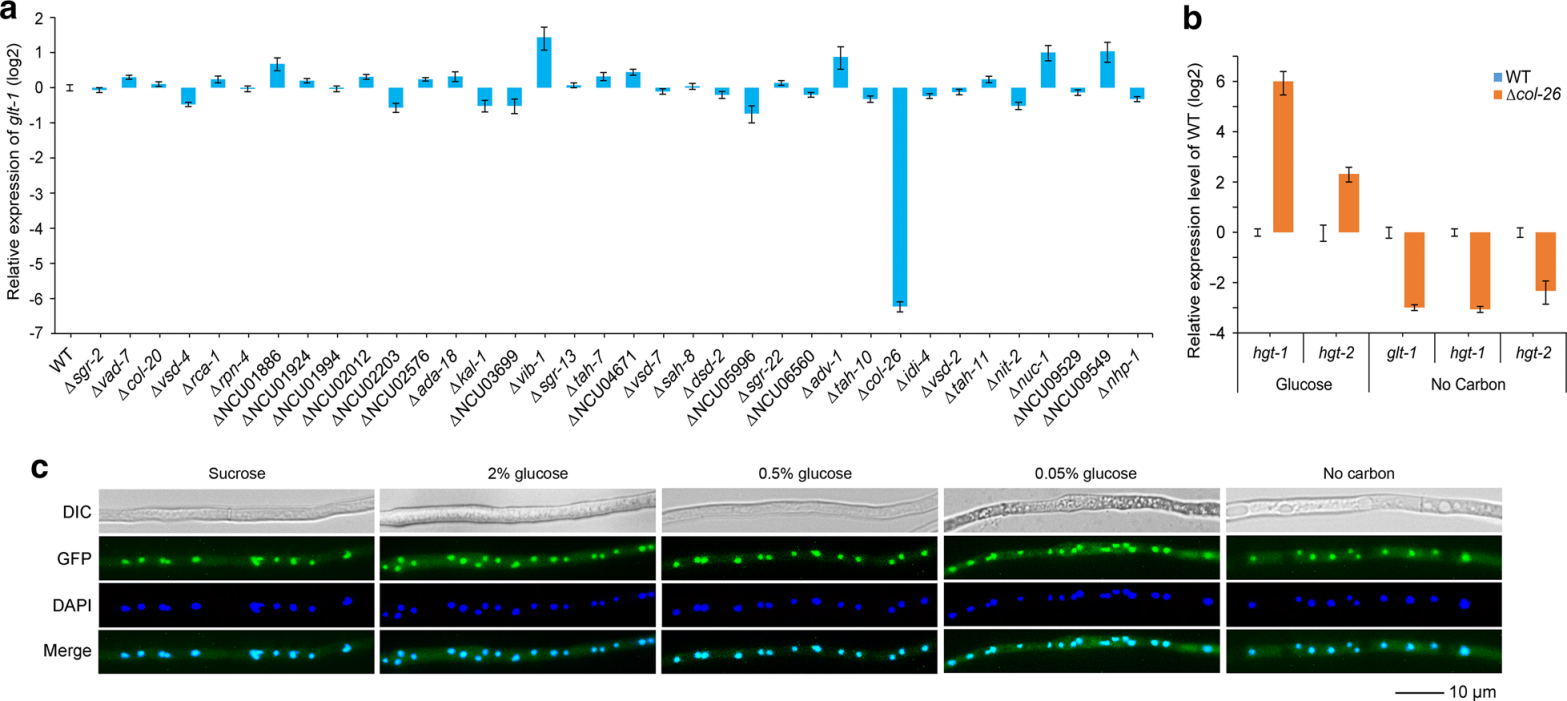
COL-26 is a key transcription factor that regulates the dual-affinity glucose transport system in *N. crassa*. **a** Relative expression level of *glt-1* in 36 transcription factor mutants of *N. crassa* grown in Vogel’s minimal medium (VMM) supplemented with glucose as the sole carbon source. Detailed information about these transcription factors is shown in Additional file 1: Table S1. **b** Relative expression of *glt-1* and *hgt-1/-2* in the presence of glucose and in carbon-free conditions in the wild-type (WT) strain and Δ*col-26* mutant. **c** Subcellular location of COL-26 in *N. crassa* exposed to a gradient of glucose. Scale bar represents 10 µm

To search for transcription factors essential for expression of *glt-1*, deletion mutants of 36 transcription factors of *N. crassa*, based on their expression in adequate glucose conditions (Additional file 1: Table S1) [51], were chosen and screened through batch culture with glucose as the sole carbon source followed by qRT-PCR assay. Most of the mutants showed no significant change in *glt-1* expression compared with the WT strain. However, the Δ*col-26* mutant showed dramatically decreased expression of *glt-1* (Fig. 1a). COL-26 is a zinc binuclear cluster [Zn(II)_2_Cys_6_] DNA-binding protein that is essential for starch utilization [69]. The abnormal expression of *hgt-1/-2* and *glt-1* in Δ*col-26* mutant compared with WT strain (Fig. 1b) suggested that COL-26 is involved in regulating the dual-affinity glucose transport system in *N. crassa*. The nuclear localization of COL-26 was independent of glucose concentration (Fig. 1c).

**Fig. 2:**
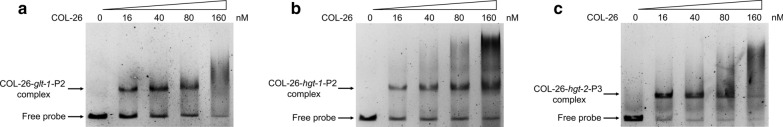
Electrophoretic mobility shift assays of binding of COL-26 to upstream DNA regions of *glt-1* (**a**), *hgt-1* (**b**), and *hgt-2* (**c**). Each lane contained 10 ng probe and the indicated amount of purified COL-26 binding domain (in nM)

To further investigate whether COL-26 directly regulates *glt-1* and *hgt-1/-2*, EMSAs were performed. GST-fused DNA-binding domain of COL-26 was expressed and purified from *E. coli* (Additional file 2: Figure S1). EMSA results showed that the recombinant COL-26 bound to the promoter regions of all three genes in a typical protein concentration-dependent manner (Fig. 2a–c).

### Δ*col-26* mutant phenotypically and transcriptionally resembles Δ*rco-3* mutant

**Fig. 3:**
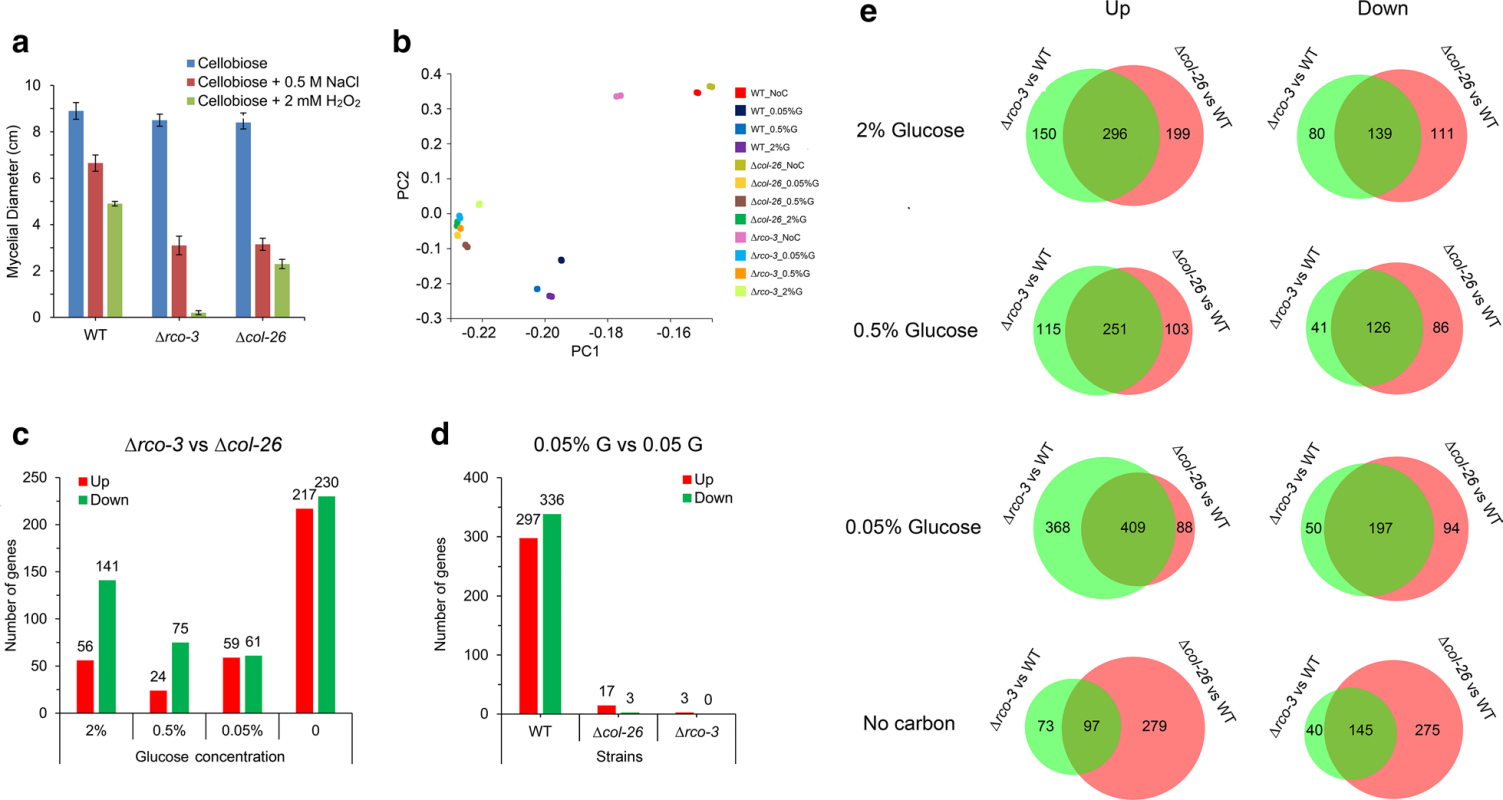
The Δ*col-26* mutant phenotypically resembles a Δ*rco-3* mutant of *N. crassa*. **a** Mycelial diameter of the WT strain, and Δ*col-26* and Δ*rco-3* mutants grown on VMM plus 2% cellobiose at 28 °C for 30 h with or without 0.5 M NaCl or 2 mM H_2_O_2_. **b** Principal component analysis of RNA-Seq data from the WT, Δ*rco-3*, and Δ*col-26* strains grown in different concentrations of glucose. **c** Number of differentially expressed genes between Δ*rco-3* and Δ*col-26* strains in each glucose condition. **d** Number of differentially expressed genes between 0.05% glucose and 0.5% glucose for the WT, Δ*rco-3*, and Δ*col-26* strains. **e** Venn diagram of differentially expressed genes in the Δ*rco-3* mutant and Δ*col-26* mutant compared with the WT in different concentrations of glucose

In *N. crassa*, RCO-3 acts as a non-transporting glucose sensor [67]. Expression of *glt-1* is dramatically downregulated in a Δ*rco-3* mutant, whereas the expression of *hgt-1/-2* is significantly upregulated in glucose-rich conditions [51], which is similar to the Δ*col-26* mutant (Fig. 1a, b). In addition, both mutants are defective in glucose uptake and biomass accumulation in the presence of high glucose levels, and are resistant to 2-deoxyglucose (2-DG, which cannot be catalyzed during glycolysis and is a drug often used for glucose repression analysis in filamentous fungi) [4, 67]. Also, both mutants were sensitive to osmotic stress and H_2_O_2_-induced oxidative stress compared with the WT strain, as shown by plate growth assays (Additional file 3: Figure S2) and the corresponding mycelial diameter of the Δ*col-26* and Δ*rco-3* mutants (Fig. 3a). We assumed that COL-26 and RCO-3 are probably in a common regulatory cascade, in which membrane-located RCO-3 transduces a glucose signal to nuclear-located COL-26 in the presence of glucose.

To test this hypothesis and also obtain a broad view of the mode of expression, we conducted high-throughput sequencing (RNA-Seq) of wild-type, Δ*col-26*, and Δ*rco-3* mycelia exposed to a gradient of glucose (0, 0.05, 0.5, 2.0%) for 1 h. Pearson and Spearman correlation analysis demonstrated that the biological replicates were reliable for all tested samples (Additional file 4: Figure S3a). RNA-Seq data (Additional file 1: Table S2) from the WT, Δ*col-26*, and Δ*rco-3* biological replicates were subjected to principal component analysis and data from the same strain grown in the same growth conditions clustered together. Compared with the WT strain, data from the Δ*col-26* mutant and the Δ*rco-3* mutant exposed to glucose (0.05, 0.5 and 2.0%) clustered together (Fig. 3b). This indicated that both mutants had impaired transcriptomic responses to 0.05, 0.5, and 2% glucose and had similar expression profiles, which was in accordance with sample-to-sample clustering (Additional file 4: Figure S3b). Consistent with these observations, the number of differentially expressed genes (DEGs) in Δ*rco-3 vs.* Δ*col-26* was much lower than that in Δ*rco-3 vs.* WT and Δ*col-26 vs.* WT exposed to glucose (Fig. 3c and e, Additional file 1: Table S3). In addition, the number of DEGs in the Δ*rco-3* mutant and Δ*col-26* mutant comparing 0.05% glucose with 0.5% glucose was dramatically lower than the number in the WT (Fig. 3d, Additional file 1: Table S3). Further investigation showed that in the presence of glucose, *rco-3* and *col-26* regulate a large proportion of DEGs in common as described above, whereas there were far fewer DEGs in Δ*rco-3 vs.* WT than in Δ*col-26 vs.* WT in carbon-free conditions, or in Δ*rco-3 vs.* WT in glucose conditions (Fig. 3e, Additional file 1: Table S3). This indicated that COL-26 functions in both glucose and carbon-free conditions, whereas RCO-3 mainly functions in the presence of glucose.

Next, the effect of COL-26 and RCO-3 on the sugar uptake system was investigated. Among the 39 putative sugar transporters present in the genome of *N. crassa* [70], 26 showed robust expression levels (FPKM > 20) in at least one condition (Additional file 5: Figure S4). In the WT strain, the transcriptional responses of these sugar transporters to a glucose gradient were in good accordance with previously published data [51]. Some genes displayed a strong or moderate response to external glucose changes, including *glt-1*, *hgt-1/-2*, *xyt-1*, *cdt-1/-2*, NCU05897, NCU00821, *xat-1*, *lat-1*, *gat-1*, *clp-1*, and NCU09287. However, the responses of these transporter genes to a glucose gradient (2, 0.5, and 0.05%) were impaired in Δ*rco-3* and Δ*col-26* mutants (Additional file 5: Figure S4). This is consistent with the observations that both mutants had similar transcriptional profiles and impaired transcriptomic response to glucose fluctuation (Fig. 3b–e). As for *glt-1*, *hgt-1/-2*, and *xyt-1*, which displayed the strongest response to external glucose changes in the WT strain, their changed expression was probably due to the absence of COL-26 in the Δ*col-26* mutant or an inactivated form of COL-26 in the Δ*rco-3* mutant. Significantly downregulated expression of *glt-1* was observed in both the Δ*col-26* and Δ*rco-3* mutants at all glucose concentrations, even though the expression level of *col-26* in the Δ*rco-3* mutant was almost three times that in the WT strain in the presence of 2 and 0.5% glucose (Additional file 6: Figure S5 and Additional file 1: Table S2), indicating that both genes are essential for *glt-1* expression and that RCO-3 acts upstream of COL-26. Notably, *hgt-1* and *hgt-2* had very similar expression profiles (Additional file 5: Figure S4), indicating the synergetic regulation of the two major components of the high-affinity glucose transport system [51].

### Phosphoproteome of the WT strain grown on glucose *vs.* starvation conditions

**Fig. 4:**
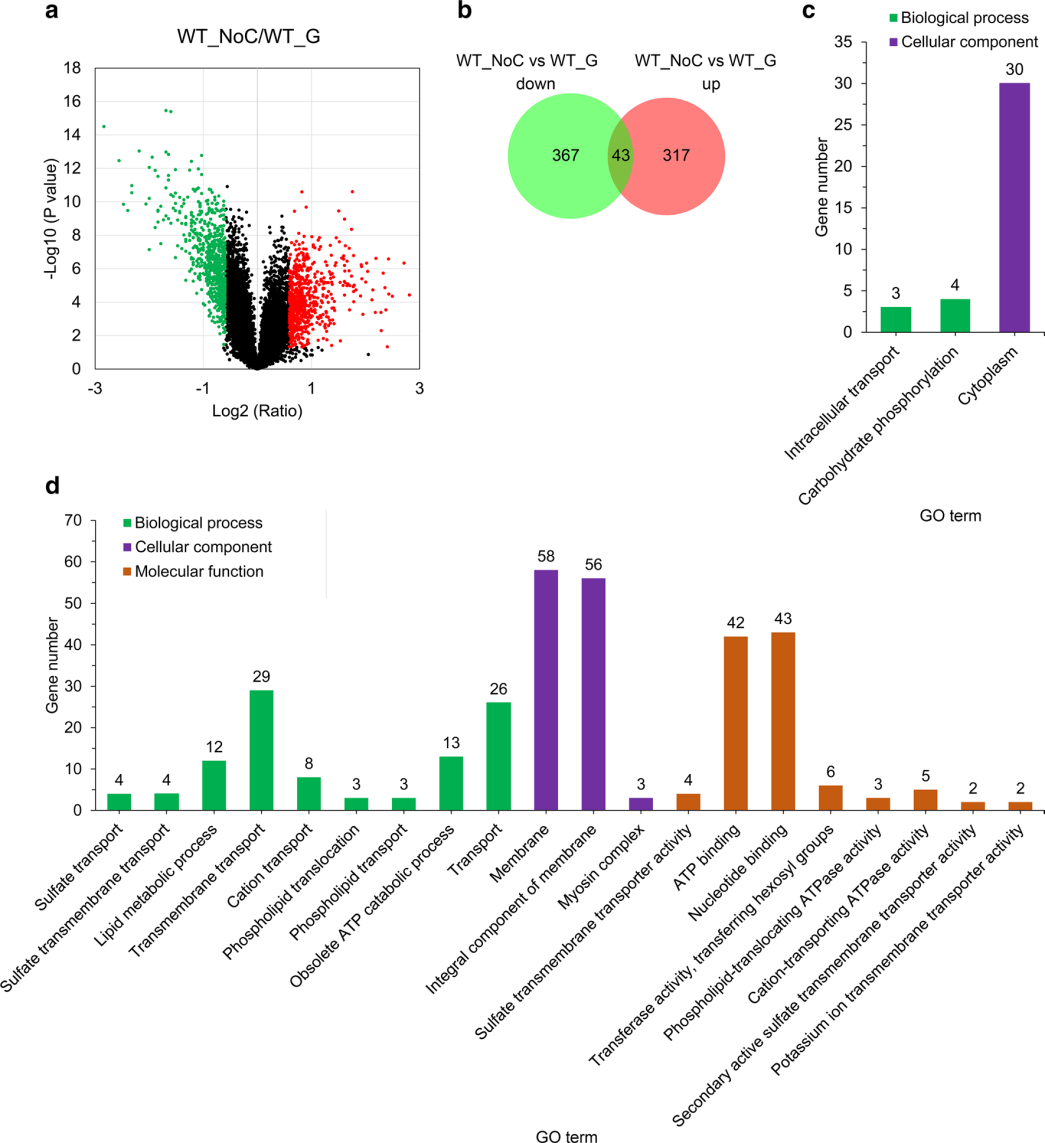
Phosphoproteomic analysis of the WT strain of *N. crassa* grown on glucose or in no-carbon (NC) conditions for 1 h. **a** Differential phosphopeptides between NC and glucose (Glu) conditions. **b** Venn diagram of proteins whose peptides showed differential phosphorylation levels. **c** Gene Ontology (GO) enrichment analysis of the 317 proteins highly phosphorylated in the comparison NC *vs*. Glu. **d** GO enrichment analysis of the 367 proteins highly dephosphorylated in the comparison NC *vs*. Glu

Given that expression of *col-26* at the transcriptional level is not affected by a gradient of glucose (0–10% w/v) [51], we wondered if a post-translational modification, such as the phosphorylation level of COL-26, explains the significant differentially expression of *glt-1* between carbon-rich (2% glucose) and starvation (no-carbon, NC) conditions. Phosphoproteome profiling of the WT strain grown on glucose (Glu) compared with starvation conditions was performed. The coefficient of variation showed that the phosphopeptide abundance correlated well between the three replicates in each condition (Additional file 7: Figure S6). We identified 11,992 phosphopeptides, mapped to 2508 proteins (Additional file 1: Table S4). Of these phosphopeptides, 661 (representing 360 proteins) increased in abundance, and 709 (representing 410 proteins) decreased in abundance in the NC *vs.* Glu comparison (Fig. 4a and Additional file 1: Table S5).

There are 43 proteins highly phosphorylated in one or more regions, but dephosphorylated in other regions in the NC *vs.* Glu comparison (Fig. 4b), including protein phosphatase regulator REG1, nitrate nonutilizer-2 NIT-2, eukaryotic peptide chain release factor ERF2, chromatin remodeling factor CRF4-3, and the S/T protein kinases STK-10, STK-30, and STK-31. A similar phenomenon was also observed by Xiong et al*.* [71]. GO analysis showed that proteins highly phosphorylated on starvation *vs*. Glu were over-represented in the categories cytoplasm (GO: 0005737) (30), intracellular transport (GO:0046907) (3), and carbohydrate phosphorylation (GO:0046835) (4) (Fig. 4c and Additional file 1: Table S6), and included some glycolytic proteins such as two hexokinases (EMP-1 and NCU00575), two 6-phosphofructo-2-kinases (NCU01178 and NCU01728), glyceraldehyde 3-phosphate dehydrogenase (GPD-1), and alcohol dehydrogenase-1 (ADH-1). Highly phosphorylated proteins not belonging to these GO terms included pyruvate dehydrogenase E1 component *α* subunit (ACE-2), transketolase (NCU01328), GLT-1, and 6-phosphogluconate dehydrogenase (PPM-2) (Additional file 1: Table S5), indicating that the glycolytic pathway and pentose phosphate pathway are regulated by post-translational modifications.

Proteins highly dephosphorylated in the NC *vs*. Glu comparison were over-represented in various categories mainly associated with the membrane, transport, and ATP metabolism (Fig. 4d and Additional file 1: Table S6), suggesting active metabolism in the presence of glucose. Pathway enrichment analysis of the highly dephosphorylated proteins using the Kyoto Encyclopedia of Genes and Genomes (KEGG) identified only one pathway––the MAPK signaling pathway-yeast (ko04011)—including protoperithecium-1 (PP-1), osmotic sensitive-4 (OS-4), MAPKK kinase NRC-1, an uncharacterized protein (NCU06252), WSC-1, osmotic sensitive-2 (OS-2), and mitogen-activated protein kinase-2 (MAK-2). NRC-1 (MAPKKK) and MAK-2 (MAPK) are core components of the conserved *N. crassa* MAK-2 pathway [72] that mediates cell fusion and activates transcription factor PP-1 required for the activation of genes that play a role during the cell fusion [73]. However, another core component, MEK-2 (MAPKK), was highly phosphorylated in the NC *vs*. Glu comparison (Additional file 1: Table S5). Other highly dephosphorylated proteins in dataset NC *vs*. Glu included a scaffold protein HAM-5 of the MAK-2 pathway, HAM-8, HAM-9, CSP-6, RCO-1, ADA-3, and PRK1, which all relate to the NRC-1/MEK-2/MAK-2 signaling pathway and are required for cell-to-cell fusion [74, 75]. OS-4 (MAPKKK) and OS-2 (MAPK) are components of the hyperosmotic response (OS) MAP kinase pathway involved in carbon sensing [76]. Other highly dephosphorylated peptides in the NC *vs*. Glu comparison were from CK-1b, which is involved in growth and developmental processes [77]; ASD-4, which functions in ascus and ascospore development [78]; CEL-2, which is involved in fatty acid biosynthesis [79]; an actin-binding protein FIM [80]; and COL-26. Three phosphopeptides from COL-26 showed S79 and S83 phosphorylation decreased in abundance in the NC *vs.* Glu comparison and no other phosphorylated sites were found in COL-26 (Additional file 1: Table S5).

### The function of COL-26 itself may be regulated at the protein level rather than the phosphorylation level

**Fig. 5:**
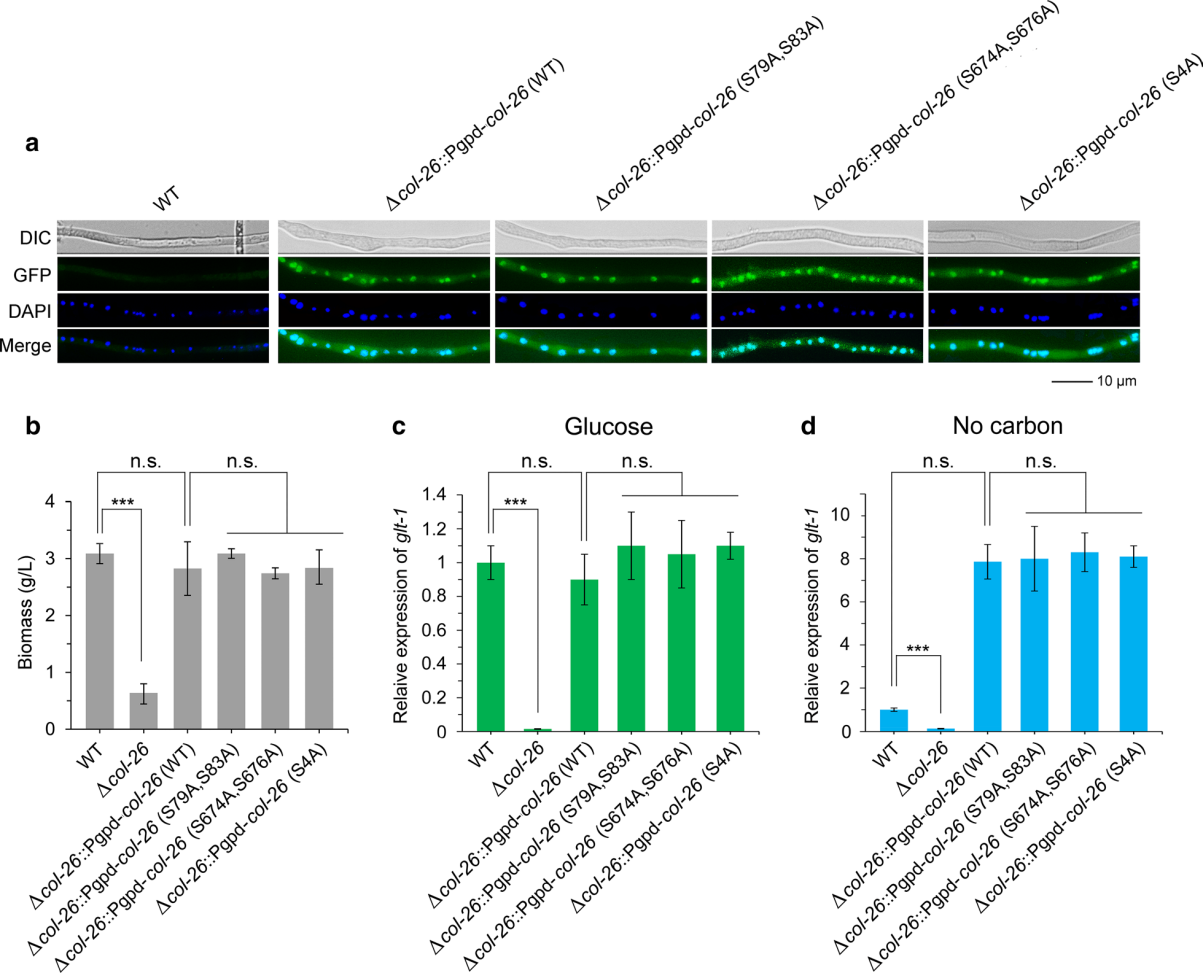
Determination of the role of residues S79, S83, S674, and S676 of *N. crassa* COL-26 in regulation of the dual-affinity glucose transport system. **a** Subcellular location of WT and point-mutated COL-26. Strains were grown in liquid VMM supplemented with 2% sucrose for 16 h at 28 °C. Scale bar represents 10 µm. **b** Biomass of different strains grown in liquid VMM containing 2% sucrose for 16 h. **c** Relative expression levels of *glt-1* in different strains in glucose condition. **d** Relative expression levels of *glt-1* in different strains in carbon-free condition. Mycelia were grown in VMM plus 2% sucrose for 16 h, then transferred to VMM with (**c**) or without (**d**) 2% glucose. After additional cultivation for 1 h, mycelia were harvested and mRNA was extracted, after which quantitative real-time qPCR was performed to determine *glt-1* expression. *n.s.* not significant; ***P < 0.001

Previous studies have identified four phosphorylation sites (S79, S83, S674, and S676) in COL-26 [71, 72, 81], among which S79 and S83 were also identified in this study and showed decreased abundance in the NC *vs*. Glu comparison (Additional file 1: Table S5). To dissect potential functions of these phosphorylation sites in the expression of the dual-transporter system, we constructed plasmids harboring *col-26-egfp* without or with site-directed mutations under the control of the promoter of the glyceraldehyde-3-phosphate dehydrogenase-1 gene (*gpd-1*). Nuclear localization of WT COL-26 and protein with simultaneous mutations at S79 and S83 (S79A, S83A), S674 and S676 (S674A, S676A), or all four sites (S4A) (Fig. 5a), as well as their recovered biomass relative to Δ*col-26* mutant on culture grown with sucrose (Fig. 5b), indicated the successful expression and correct function of these analogs. Expression of *glt-1* in Δ*col-26*::P*gpd*-*col-26* (S79A, S83A), Δ*col-26*::P*gpd*-*col-26* (S674A, S676A), and Δ*col-26*::P*gpd*-*col-26* (S4A) in glucose and NC conditions was not different from that in Δ*col-26*::P*gpd*-*col-26* (WT) (Fig. 5c and 5d), suggesting that these phosphorylation sites of COL-26 are not involved in the regulation of glucose transporter expression in *N. crassa*.

**Fig. 6:**
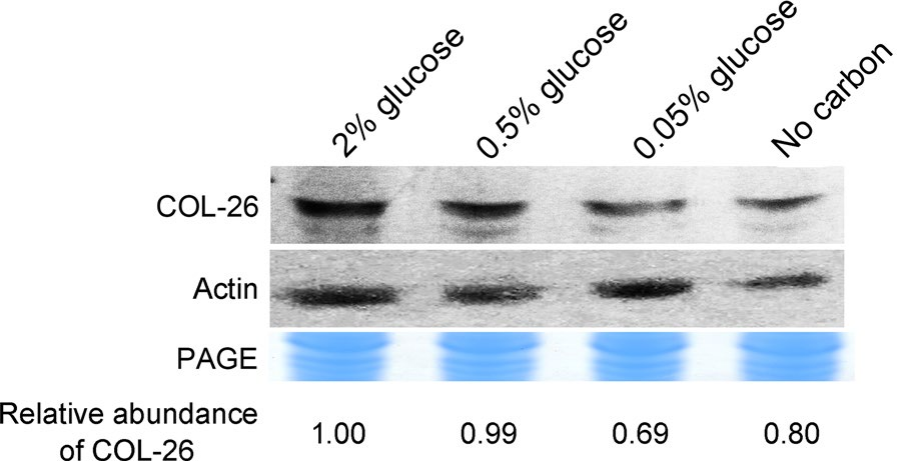
Western blotting analysis of COL-26 in the WT strain of *N. crassa*. Mycelia were cultivated in VMM supplemented with 2% sucrose for 16 h, then transferred to VMM supplemented with a gradient of glucose for an additional 1 h. The same amount of total protein was loaded into each lane

Notably, *glt-1* expression in the Δ*col-26* mutant complemented with WT and mutated COL-26 grown in starvation conditions was much higher than that in the WT strain (Fig. 5d), probably because of the high expression level of *col-26* driven by the *gpd-1* promoter which leads to a high level of COL-26. So, we constructed the complemented strain Δ*col-26*::Pn-*col-26* expressing *col-26-egfp* under the control of its native promoter. The protein level of COL-26 in the presence of different concentrations of glucose was determined by western blotting using anti-GFP antibody. The protein level of COL-26 in the presence of adequate glucose (0.5 and 2%) was higher than that in starvation conditions (0.05%, or no glucose) (Fig. 6), suggesting that the function of COL-26 itself might be regulated at the protein level rather than by phosphorylation.

### AMPK represses *glt-1* expression, possibly by inhibiting RCO-3 activity, in starvation conditions

Previous study showed that the OS MAP kinase pathway is involved in carbon sensing [76]. Two of its components (OS-2 and OS-4) were highly phosphorylated in the WT strain in the Glu *vs*. NC comparison (Additional file 1: Table S5). Thus, the roles of this pathway in regulation of the dual-affinity glucose transport system were investigated by characterization of deletion mutants of *os-1* and *os-2*, two essential components of this pathway. Expression of *glt-1* and *hgt-1* in Δ*os-1* and Δ*os-2* mutants showed no difference from that in the WT strain in either glucose-rich or starvation conditions. *glt-1* and *hgt-1/-2* expression in Δ*rco-3;*Δ*os-1* and Δ*rco-3;*Δ*os-2* double mutants was identical to that in the Δ*rco-3* mutant in both conditions (Additional file 8: Figure S7). These results indicate that *os-1* and *os-2* are not involved in regulation of the dual-affinity glucose transport system.

**Fig. 7:**
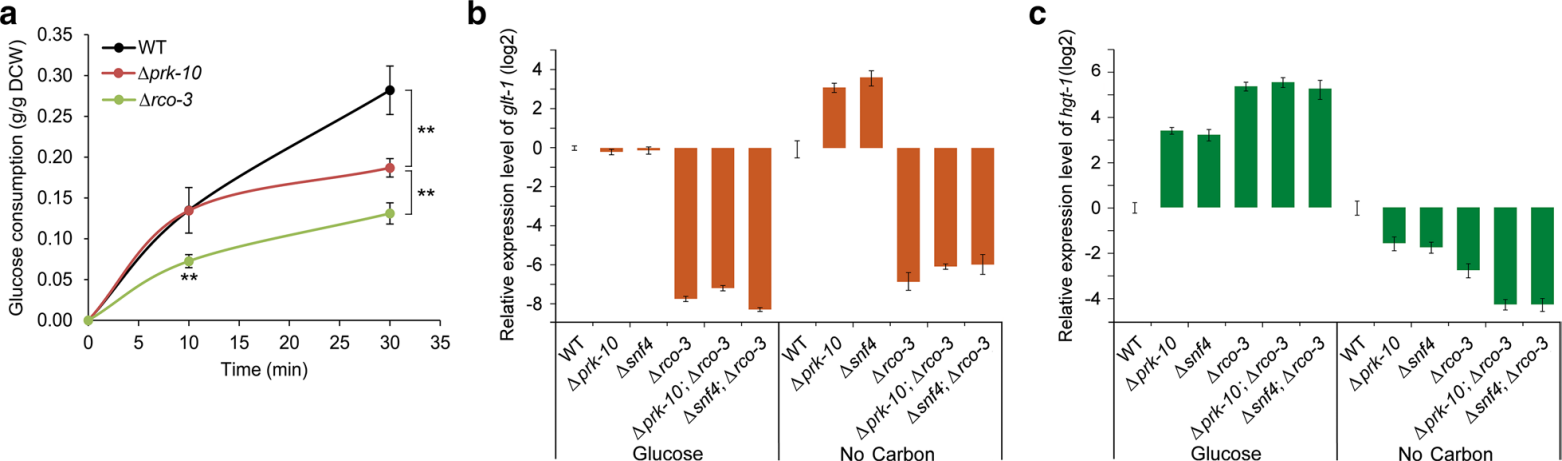
AMPK complex involves in regulation of glucose dual-affinity transport system. **a** Glucose uptake of WT, Δ*prk-10*, and Δ*rco-3* mutants in low glucose concentration. **P < 0.01. **b** Relative expression levels of *glt-1* in WT, Δ*prk-10*, Δ*snf4*, Δ*rco-3*, Δ*rco-3*;Δ*prk-10*, and Δ*rco-3*;Δ*snf4* strains of *N. crassa* in glucose and no-carbon conditions. **c** Relative expression levels of *hgt-1* in WT, Δ*prk-10*, Δ*snf4*, Δ*rco-3*, Δ*rco-3*;Δ*prk-10*, and Δ*rco-3*;Δ*snf4* strains in glucose and no-carbon conditions. Mycelia were grown in VMM supplemented with 2% sucrose for 16 h, then transferred to VMM with or without 2% glucose. After additional cultivation for 1 h, mycelia were harvested and the *glt-1* expression level was determined by qRT-PCR

In *S. cerevisiae*, the SNF1 complex plays an important role in regulation of glucose transport [33, 38]. AMP-activated protein kinase (AMPK) complex from higher eukaryotes is homologous to SNF1 complex and mainly functions in nutrient-limited condition to maintain cellular energy homeostasis. In *N. crassa*, *prk-10* (the ortholog of *snf1*) and NCU01471 (here named as *snf4*) encode the *α*-subunit and *γ*-subunit of the AMPK complex, respectively. To test whether AMPK involves in glucose transport, we measured glucose uptake rates in WT, the Δ*prk-10*, and the Δ*rco-3* mutants in low glucose concentration. Within the first 10 min, the Δ*prk-10* mutant consumed the same amount of glucose as WT strain. However, over the remaining 20 min, glucose uptake rates decreased dramatically in the Δ*prk-10* mutant (Fig. 7a). The Δ*rco-3* mutant, as expected, showed decreased glucose uptake rates all the time (Fig. 7a). To further investigate the role of AMPK in glucose transport, the effect of AMPK on expression of *glt-1* was investigated. Expression of *glt-1* in Δ*prk-10* and Δ*snf4* mutants was the same as that in the WT strain in the presence of glucose, but significantly upregulated in starvation conditions. Expression of *glt-1* in Δ*rco-3;*Δ*prk-10* and Δ*rco-3;*Δ*snf4* double mutants was the same as that in the Δ*rco-3* mutant (Fig. 7b), indicating that AMPK-repressed expression of *glt-1* might occur via inhibition of RCO-3 activity in starvation conditions. This conclusion was supported by transcriptomic data, which showed that *rco-3* mainly functions in the presence of glucose (Fig. 3e). Notably, the lower number of DEGs in the Δ*rco-3 vs.* WT comparison in starvation conditions compared with glucose condition (Fig. 3e) and the significantly reduced expression level of *glt-1* in the Δ*rco-3* mutant (Fig. 7b) indicated that RCO-3 activity was not totally inhibited in starvation condition. Though deletion of *prk-10* or *snf4* had no effect on *glt-1* expression in glucose-rich conditions, expression of *hgt-1* was upregulated when the Δ*prk-10* and Δ*snf4* mutants were grown on glucose (Fig. 7c). Besides, deletion of *prk-10* or *snf4* in the Δ*rco-3* background further decreased *hgt-1* expression in starvation conditions (Fig. 7c), indicating that other regulatory component(s) are also involved in regulation of *hgt-1* expression.

### The glucose transport system shows conserved regulation by COL-26-like transcription factors in ascomycete species

**Fig. 8:**
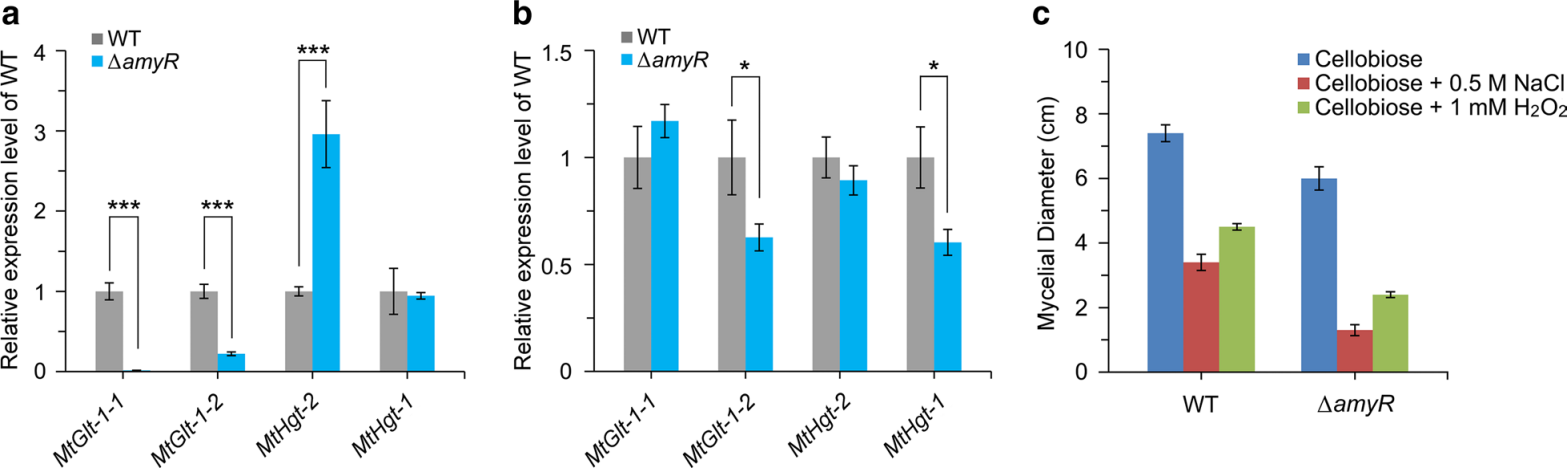
Phenotype of *Myceliophthora thermophila* Δ*amyR* mutant. **a** Expression of glucose transporter genes in the presence of glucose. **b** Expression of glucose transporter genes in no-carbon conditions. *P < 0.05; ***P < 0.001. **c** Mycelial diameter of the WT strain and Δ*amyR* mutant grown on VMM plus 2% cellobiose at 37 °C for 4 days with or without 0.5 M NaCl or 1 mM H_2_O_2_

Despite some minor differences in use of some kinds of sugars, conserved roles of COL-26/AmyR homologs have been reported in various fungi, including *Magnaporthe oryzae* [82], *Fusarium graminearum* and *F. verticillioides* [83], *A. nidulans* [84], *A. oryzae* [68], *A. niger* [85], *T*. *reesei* [86], *Penicillium oxalicum* [87], *Talaromyces pinophilus* [88], and *Myceliophthora thermophila* [89]. In *P. oxalicum*, the *N. crassa* HGT-1 ortholog PDE_03475 showed a high expression level on cellulose and a decreased expression level in a Δ*amyR* mutant compared with the WT strain [87]. In *A. niger*, the *A. nidulans* MstE ortholog An02g03540 showed a high expression level on glucose and maltose, and its expression in a Δ*amyR* mutant was significantly downregulated [90]. To test the hypothesis that the dual-affinity glucose transport system and its regulation by COL-26 homologs are conserved in filamentous fungi, the effect of deletion of *M. thermophila* AmyR, the closest homolog to *N. crassa* COL-26, on expression of the putative dual-affinity glucose transport system was investigated. The *M. thermophila* Δ*amyR* mutant exhibited significantly reduced growth on glucose, fructose, sucrose, maltose, trehalose, xylose, and soluble starch, but grew well on cellobiose and cellulose [89], which is similar to the *N. crassa* Δ*col-26* mutant. Alignment showed that Mycth_112491 (named as MtGLT-1-1) is the closest ortholog of *N. crassa* GLT-1. However, MtGLT-1-1 is only 352 amino acids long with six transmembrane helices (TMHs), compared with the typical 12 TMHs for glucose transporters predicted by TMHMM Server v2.0. The second closest GLT-1 ortholog is Mycth_108924 (named as MtGLT-1-2), which has 12 predicted TMHs. Both *MtGlt-1-1* and *MtGlt-1-2* showed an elevated expression level on glucose compared with NC and cellulose conditions [89, 91]. Mycth_2308157 (named as MtHGT-1) and Mycth_2295230 (named as MtHGT-2) are the closest orthologs of *N. crassa* HGT-1 and HGT-2, respectively. Both *MtHgt-1* and *MtHgt-2* showed increased expression on cellulose compared with glucose [91], while *MtHgt-2* also showed significantly higher expression in NC conditions than in glucose-rich conditions [89], consistent with the expression pattern of high-affinity glucose transporters. These data were supported by qRT-PCR (Additional file 9: Figure S8a). We determined the expression levels of these glucose transporter-encoding genes in a Δ*amyR* mutant of *M. thermophila*. In glucose-rich conditions, both *MtGlt-1-1* and *MtGlt-1-2* showed significantly decreased expression, whereas *MtHgt-2* was upregulated in the Δ*amyR* mutant, compared with the WT strain (Fig. 8a). In NC conditions, *MtGlt-1-2* and *MtHgt-1* showed decreased expression levels in the Δ*amyR* mutant compared with WT strain (Fig. 8b). This indicates that AmyR is a key transcription factor involved in regulation of the dual-affinity glucose transport system in *M. thermophila*, like COL-26 in *N. crassa*. In addition, like the Δ*col-26* mutant of *N. crassa*, the Δ*amyR* mutant of *M. thermophila* was sensitive to osmotic stress and H_2_O_2_-induced oxidative stress compared with the WT strain, as shown by plate growth assays (Additional file 9: Figure S8b) and the corresponding mycelial diameter (Fig. 8c). Since COL-26 orthologs and the dual-affinity glucose transporter system are ubiquitous in ascomycete species based on phylogenetic analysis [51, 69], the regulatory role of *col-26* orthologs may also be conserved in many other filamentous fungal species.

## Discussion

Glucose uptake is the first and rate-limiting step of glucose metabolism. To cope with environmental changes in glucose availability, fungi express low-affinity glucose transporters when glucose levels are high and high-affinity glucose transporters when glucose levels are low. The *N. crassa* dual-affinity glucose transport system is the best-studied example, and consists of a low-affinity glucose transporter GLT-1 and two high-affinity glucose transporters HGT-1 and HGT-2 [51]. This dual-transporter system is conserved in filamentous fungi and many glucose transporters have been characterized. However, the mechanism underpinning the regulation of the dual-affinity glucose transport system in response to environmental changes remains elusive. Here, using *N. crassa* as a model fungus, we identified a glucose signaling pathway consisting of multiple components, including the major transcription factor COL-26, non-transporting glucose sensor RCO-3, and a cellular energy sensor AMPK. COL-26 regulates the dual-affinity glucose transport system at the transcriptional level. Deletion of *col-26* or *rco-3* leads to a significantly reduced expression level of *glt-1* in both glucose-rich and starvation conditions (Figs. 1a and 7b), indicating that the basal level of *glt-1* in starvation conditions is also maintained by COL-26 and RCO-3. Interestingly, we found that COL-26-dependent regulation of the dual-transporter system might depend on the regulation of its protein homeostasis, rather than on post-translational modification such as phosphorylation. The expression level of *col-26* in the WT strain remains the same in glucose-rich and carbon-free conditions, and it is only induced by starch, trehalose and maltose [51, 69] (Additional file 1: Table S2). This could reduce the transcriptional and translational investment when *N. crassa* growing in nutrition-deficient conditions encounters nutrient-rich conditions. Upon nutrition shift, the basal level *glt-1* mRNA can be immediately translated, and there is no need to synthesize *col-26* mRNA de novo for translation. Once COL-26 is synthesized from existing mRNA, it promotes expression of *glt-1* in a COL-26 concentration-dependent manner at the protein level (Fig. 6) rather than in a phosphorylation-dependent manner (Fig. 5). However, overexpression of *col-26* did not further promote expression of *glt-1* in the presence of glucose (Fig. 5c), indicating that COL-26 does not function independently. Other components of the pathway that control COL-26 synthesis/degradation or dissociation/association with other protein(s) are worth investigating in the future. Moreover, as only four phosphorylation sites (S79, S83, S674, and S676) have so far been identified in COL-26 by various studies [71, 72, 81] (Additional file 1: Table S4), whether other phosphorylation site(s) are involved in the regulation of the dual-affinity glucose transport system requires investigation.

The detailed mechanism of regulation of glucose dual-affinity transport system by the key transcription factor COL-26 under different glucose conditions needs to be further investigated. Some explanation could be drawn from a previous study which showed that the absence of GLT-1 leads to the upregulated expression of *hgt-1/-2* in high glucose condition [51]. This indicates that the upregulated expression of *hgt-1/-2* in Δ*glt-1* mutant was due to relief from CRE-1-mediated CCR which is caused by its inability of transporting high amount of glucose. Since *glt-1* expression was abolished in Δ*col-26* and Δ*rco-3* mutant, the upregulated expression of *hgt-1/-2* in Δ*col-26* and Δ*rco-3* mutant in response to glucose was probably also caused by relief from CCR, similar to the Δ*glt-1* mutant.

**Fig. 9:**
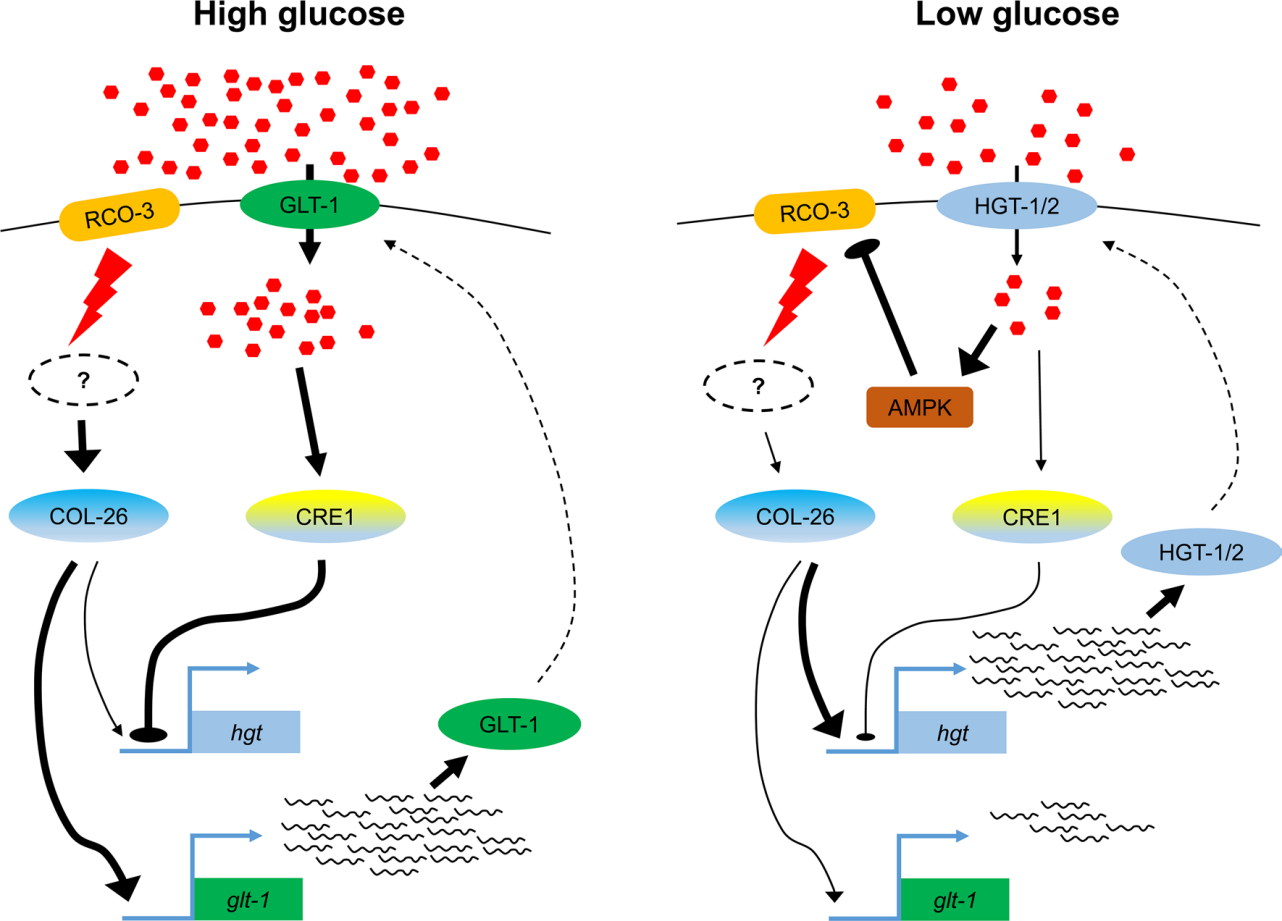
Model for the role of COL-26 in regulating the dual-affinity glucose transport system in filamentous fungi. Under high levels of glucose, RCO-3 transduces a glucose signal to COL-26 to promote expression of *glt-1* for nutrient assimilation. Meanwhile, adequate glucose stimulates CRE-1-mediated carbon catabolite repression (CCR) to repress expression of *hgt-1/-2*. When external glucose is depleted or limited, *hgt-1* and *hgt-2* are rapidly derepressed by the lifting of CCR and their expression is activated by COL-26. This process synergistically activates AMP-activated protein kinase (AMPK), which inhibits activity of RCO-3, leading to low expression of *glt-1*

The high expression level of *hgt-1*/*-2* in glucose-limited conditions is dependent on COL-26 (Fig. 1b), however, expression of *hgt-1*/*-2* in the presence of adequate glucose was repressed by CRE-1-mediated CCR [51], even though COL-26 remains at a relatively high level (Fig. 6), indicating that CRE-1 could be antagonistic to COL-26. Therefore, the high expression level of *hgt-1*/*-2* in glucose-limited conditions is due to a combination of derepression from CRE-1 and activation by COL-26 (Fig. 9). Taken together, we proposed that COL-26 and CRE-1 probably binds to the same promoter regions of *hgt-1/-2*. In WT strain under glucose-rich condition, CRE-1 binds to promoter regions of *hgt-1/-2* to repress their expression. Deletion of *col-26* leads to abolishment of GLT-1 and subsequent impaired glucose transport, leading to relief from CCR and disassociation of CRE-1 from their promoters, thus derepressing the expression of *hgt-1/-2*; whereas, under starvation condition, after disassociation of CRE-1 from *hgt-1/-2* promoters, COL-26 might bind to the same region to activate their expression. When *col-26* was deleted, this activation was absent, thus leading to the downregulation of *hgt-1/-2* (Fig. 1b). The hypothesis needs to be investigated through further studies.

Another finding of this study is that RCO-3 is deeply involved in the regulation of the glucose transport system, since the expression of low-affinity glucose transporter gene *glt-1* under starvation was repressed by AMPK through partially inhibiting the activity of RCO-3 (Fig. 7b). The similarity of multiple phenotypes between the Δ*col-26* mutant and Δ*rco-3* mutant indicates that RCO-3 and COL-26 are in the same signaling pathway, in which RCO-3 acts as a glucose sensor and transduces a glucose signal to nuclear COL-26. Supporting this, the expression level of *col-26* in the Δ*rco-3* mutant was almost three times that in the WT strain in the presence of a high glucose concentration (Additional file 6: Figure S5 and Additional file 1: Table S2), but expression of *glt-1* was still significantly downregulated due to the absence of RCO-3 (Fig. 7b). Besides, components downstream of RCO-3 and upstream of COL-26, but not yet identified, probably regulate not only the dual-affinity glucose transport system, but also other biological processes. Identification of the missing part(s) of the RCO-3/COL-26 signaling pathway will help us to understand the architecture of nutrient signaling regulation in filamentous fungi.

## Conclusions

In this study, we identified the crucial transcription factor COL-26 that regulates the glucose dual-affinity transport system by directly binding their promoter region in *N. crassa*. COL-26 is downstream element of the non-transporting glucose sensor RCO-3. In addition, the AMPK complex is required to suppress the expression of low-affinity glucose transport system in starvation condition by partially inhibiting the activity of RCO-3. Knowledge about regulatory mechanism of the dual-transporter system will not only deepen our understanding of the environmental adaptability of filamentous fungi, but also aid in the rational design of fungal strains for industrial purposes.

## Methods

### Strains

*Escherichia coli* strains DH5*α* (Invitrogen, Shanghai, China) and BL21 (DE3; Gibco BRL, Rockville, MD, USA) were used for plasmid propagation and gene expression, respectively. *M. thermophila* ATCC 42464 was obtained from the American Type Culture Collection. A Δ*amyR* mutant of *M. thermophila* was constructed by our laboratory in a previous study [89]. Strains of *N. crassa* were obtained from the Fungal Genetics Stock Center (FGSC, http://www.fgsc.net), including the wild-type (WT) reference strain (FGSC 2489), Δ*col-26* (FGSC11030, *mat a*), Δ*rco-3* (FGSC17928, *mat a*), Δ*prk-10* (FGSC12421, *mat A*), and Δ*snf4* (FGSC13236, *mat A*). The double-deletion strains Δ*rco-3*;Δ*snf4* and Δ*rco-3*;Δ*prk-10* were generated by performing sexual crosses as previously described (http://www.fgsc.net/Neurospora/NeurosporaProtocolGuide.htm). The mis-expression strains were constructed by transforming the Δ*col-26* mutant with linearized plasmid pCSR1 [92] harboring the promoter of the glyceraldehyde-3-phosphate dehydrogenase-1 gene (*gpd-1*), various gene-coding sequences or point-mutated analogs, and flanking regions of the *csr-1* gene sequence. Transformants were selected for resistance to cyclosporin A and tested for genotypes by diagnostic PCR.

### Culture conditions

*E. coli* was grown at 37 °C in Luria–Bertani medium supplemented with 100 µg ml^−1^ kanamycin or ampicillin when necessary. *M. thermophila* strains were cultured on Vogel’s minimal medium (VMM) [93] supplemented with 2% sucrose at 45 °C for 7–10 days to obtain conidia. *N. crassa* strains were inoculated on slants containing 3 mL VMM with 2.0% (w/v) sucrose as the sole carbon source and grown at 28 °C in the dark for 2 days, then at room temperature in constant light for 6–10 days to stimulate conidia production. Conidia were inoculated into 100 mL liquid VMM with various carbon sources at 10^6^ conidia·mL^−1^ and grown at 25 °C in constant light with shaking (200 rpm). For plate growth assays, 1 μL of conidia suspension (1 × 10^6^ conidia·mL^−1^) was plated on VMM supplemented with 2% cellobiose and cultured at 28 °C for 30 h for *N. crassa*, or 37 °C for 4 days for *M. thermophila*. NaCl and H_2_O_2_ were added to media to a final concentration of 0.5 M and 2 mM, respectively, for *N. crassa*. For *M. thermophila*, H_2_O_2_ was added to a final concentration of 1 mM.

### Medium shift experiments

Conidia were inoculated into 100 mL liquid VMM supplemented with 2.0% sucrose and grown at 28 °C and 200 rpm in constant light for 16 h. The mycelial biomass was washed with sterilized water at least five times and then transferred to 100 mL VMM with 2% glucose or with no carbon source added for 1 h before RNA extraction.

### Plasmid construction and transformation

A fragment containing 5′- and 3′-flanking regions of *csr-1* was amplified with pLC-5-F/pLC-3-R from pCSR1 [92]. The promoter of *gpd-1* was amplified from genomic DNA using primers Pgpd-NC-F/Pgpd-NC-R. The open reading frame of *col-26* was amplified using Col26-ORF-F/Col26-ORF-R from cDNA which was synthesized from total RNA using a ReverTra Ace qPCR RT Kit (Toyobo, Japan). The coding sequence of *gfp* was amplified using primers GFP-F/GFP-R from pMF272. These four fragments were assembled using the NEB Gibson Assembly Kit (New England Biolabs, USA) to give pCSR-COL-26-GFP. The variants *col-26* (S79A, S83A), *col-26* (S674A, S676A), and *col-26* (S4A) were generated by site-directed mutagenesis using PCR with high-fidelity polymerase. Transformation by electroporation was performed as described previously [92]. Transformants resistant to cyclosporin A were further confirmed by PCR and green fluorescent protein (GFP) fluorescence.

### Quantitative real-time qPCR (qRT-PCR)

qRT-PCR was performed as previously described [94]. The *actin* gene (*act*) was used as an endogenous control for *N. crassa*, and *MtAct* was used as an endogenous control for *M. thermophila*. All primers used in this study are listed in Additional file 1: Table S7. The transcript level of each gene was estimated using the 2^−ΔΔCt^ method [95]. The ratio of each gene transcript in each mutant to that in the WT strain was calculated as the relative transcript level.

### RNA sequencing and transcription expression analysis

After harvesting via vacuum filtration, mycelia were immediately homogenized in liquid nitrogen for total RNA extraction. Total RNA was isolated from frozen samples with Trizol reagent (Invitrogen, Carlsbad, CA, USA), treated with DNase I, and purified using a Qiagen RNeasy Mini Kit (Qiagen, Hilden, Germany). RNA integrity was checked by agarose gel electrophoresis and using an Agilent Bioanalyzer 2100 system (Agilent Technologies, Santa Clara, CA, USA). Qualified RNA with OD_260_/OD_280_ > 2.0 and RNA Integrity Number > 8.0 was used for RNA-Seq, which was performed using the BGISEQ-500 platform at Beijing Genomics Institute (BGI) (Shenzhen, China). All data were generated by sequencing two independent duplicate samples. Prior to read mapping, adaptors and low-quality reads were trimmed using Trimmomatic v0.36 [96]. Filtered clean reads were aligned against predicted transcripts from the *N. crassa* OR74A genome v12 [97] using Bowtie2 v2.2.5 [98]. The read counts were determined using RSEM v1.2.8 [99]. The abundance of each transcript was calculated from fragments per kilobase of transcript per million mapped reads (FPKM) values (Additional file 1: Table S2). We used fuzzy c-means clustering to group genes based on similarity between concentration-specific gene expression patterns. Fuzzy clustering was conducted using Mfuzz v2.34.0 [100]. Differential gene expression analysis was performed using the DESeq package (v1.5.1). Genes with fold-change > 2.0 (|log2 ratio|≥ 1) and DESeq *P*_adj_-value (*Q*-value) < 0.001 were considered significantly differentially expressed between different conditions or strains. To discover significantly up- and downregulated genes, only genes with relatively high transcript abundance (FPKM-value > 20 in at least one strain) were considered for further analysis. RNA-Seq data are available at the Gene Expression Omnibus under accession number GSE157186.

### Protein gel electrophoresis

Culture supernatants were mixed with 4 × SDS loading buffer and boiled for 10 min before loading onto Criterion 4–15% Tris–HCl Precast Gels (Bio-Rad). GelCode Blue Stain Reagent (Thermo Scientific) was used for gel staining.

### Expression and purification of DNA-binding domain of COL-26

A fragment encoding the DNA-binding domain of COL-26 was amplified with primers Ecol26-F/Ecol26-R using cDNA as template. After digestion with *Bam*HI and *Xho*I, this fragment was ligated into the corresponding sites of pGEX-4T-1 (GE Healthcare) to give pGEX-col-26. The plasmid was subsequently introduced into *E. coli* BL21 (DE3) for protein expression. *E. coli* BL21 (DE3) harboring pGEX-col-26 was grown at 37 °C in 100 mL LB medium supplemented with 100 μg/mL ampicillin to an *OD*_600_ of 0.6. Isopropyl *β*-D-1-thiogalactopyranoside (IPTG) was then added to a final concentration of 0.5 mM, and the cultures were incubated for an additional 3 h at 37 °C. The cells were harvested by centrifugation and resuspended in phosphate-buffered saline followed by sonication, after which the insoluble material was removed by centrifugation at 8000 *g* for 10 min. The glutathione *S*-transferase (GST)-fused protein was purified using a BeaverBeads™ GST-tag Protein Purification Kit (Beaver, China) according to the manufacturer’s manual. Protein purity was determined by Coomassie Blue staining after 12% SDS-PAGE, and protein concentration was measured by BCA assay (Thermo Scientific, Waltham, MA, US).

### Electrophoretic mobility shift assays (EMSAs)

Different DNA fragments were used as probes in gel-shift experiments. For COL-26-binding experiments, the promoter regions of *glt-1* (P1, − 1906 to − 1442; P2, − 1461 to − 1032; P3, − 1049 to − 625; P4, − 682 to − 233); *hgt-1* (P1, − 992 to − 557; P2, − 575 to − 142); *hgt-2* (P1, − 1532 to − 1094; P2, − 1114 to − 698; P3, − 720 to − 284) were obtained by PCR from the genomic DNA of WT *N. crassa* using primers shown in Additional file 1: Table S7. The PCR products were purified by electrophoresis and quantified using a NanoDrop 2000c Spectrophotometer (Thermo Fisher Scientific). The subsequent binding experiments were performed using a modified gel mobility shift assay as described previously [101]. In each EMSA, different quantities of recombinant protein were incubated with a constant amount (10 ng) of the DNA probes individually at 25 °C for 30 min. The experiments were performed at least three times. The information of genes mentioned in this study are shown in Additional file 1: Table S8.

### Phosphopeptide identification by mass-spectrometry (MS)-based analysis

Proteins were reduced with 10 mM dithiothreitol for 1 h at 56 °C and subsequently alkylated with 55 mM iodoacetamide. Samples were digested with trypsin at 1:20 enzyme-to-substrate ratio. Digested samples were desalted using C18 solid phase extraction tubes. The resulting peptide samples were concentrated and a BCA assay was performed to determine the peptide concentration and samples were diluted with nanopure water for MS analysis. Desalted peptides were labeled with 8-plex iTRAQ reagents (AB SCIEX) according to the manufacturer’s instructions. Peptide aliquots for each sample (200 mg) were dried for TiO_2_ enrichment, and used for phosphoproteome analysis; TiO_2_ enrichment of phosphopeptides followed a previously established protocol [102]. Phosphopeptide samples were analyzed using a nanoESI system (Waters NanoAcquity LC, Waters Corporation) coupled to a Q Exactive HF mass spectrometer (Thermo Fisher Scientific). Proteomics data were analyzed using a combination of Proteome Discoverer (v1.4) and Mascot (v2.3) software. The MS results were filtered based on a 5% false discovery rate and phosphoRS probability ≥ 0.75. Phosphopeptide abundance changes >1.5-fold were considered and subjected to downstream analysis.

### Western blot analysis

Western blotting was performed as previously described [103]. Anti-GFP or anti-actin rabbit antibody and anti-rabbit IgG horseradish peroxidase-conjugated antibody at a dilution of 1:3000 were used as the primary and secondary antibodies (Abmart, Shanghai, China), respectively.

### Microscopy and imaging

To localize GFP fusion proteins using microscopy, all strains were inoculated into liquid VMM supplemented with 2% sucrose and grown for 16 h at 25 °C. The hyphae were harvested, washed several times with Vogel’s salts, transferred into media containing different concentrations of glucose, and cultured for another 1 h. Microscopic observations were performed using an Olympus BX51 fluorescence microscopy system and images were processed using ImageJ software.

### Glucose uptake assays

Conidia from 10-day-old cultures were inoculated into 100 mL VMM with 2% sucrose as the carbon source. After grown at 25 °C in constant light with shaking (200 rpm) for 16 h, cultures were centrifuged at 2000 *g* for 10 min and washed in VMM without a carbon source, followed by 1-h growth in 100 mL VMM with 0.05% glucose as carbon source. Then 0.05 g glucose was added to each culture. Culture supernatants were sampled at 0, 10, and 30 min. Glucose levels were measured using HPLC with an e2695 instrument (Waters, Manchester, United Kingdom).

## Supplementary Information

**Additional file 1.** Additional tables.

**Additional file 2: Figure S1.** Purification of recombinant COL-26 binding domain.

**Additional file 3: Figure S2.** Effect of stress on the growth of WT, Δ*col-26*, and Δ*rco-3* strains of *N. crassa*. VMM with 2% (w/v) cellobiose was used. NaCl and H_2_O_2_ were added to the medium to a final concentration of 0.5 M and 2 mM, respectively. Plates were incubated at 28 °C in the dark for 30 h before imaging.

**Additional file 4: Figure S3.** Validation of RNA-Seq data for *N. crassa* in response to a glucose gradient. **a** Spearman analysis. **b** Sample-to-sample clustering.

**Additional file 5: Figure S4.** Transcriptional responses of sugar transporter genes in the WT, Δ*rco-3*, and Δ*col-26 *strains of *N. crassa* to a glucose gradient. Heatmap analysis and clustering of 26 sugar transporter genes with robust expression levels (fragments per kilobase of transcript per million mapped reads > 20) in at least one condition. Log-transformed expression values are color-coded.

**Additional file 6: Figure S5.** Expression levels of *col-26* in WT and Δ*rco-3 *mutant response to a glucose gradient.

**Additional file 7: Figure S6.** Histogram of error distribution among biological replicates of phosphoproteome in glucose-replete or no-carbon conditions.

**Additional file 8: Figure S7.** Relative expression levels of *glt-1*, *hgt-1*, and *hgt-2* in WT, Δ*os-1*, Δ*os-2*, Δ*rco-3*, Δ*rco-3*;Δ*os-1*, and Δ*rco-3*;Δ*os-2* strains of *N. crassa* in glucose-rich (**a**) and no-carbon (**b**) conditions. Mycelia were grown in VMM supplemented with 2% sucrose for 16 h, then transferred to VMM with or without 2% glucose. After additional cultivation for 1 h, mycelia were harvested and gene expression levels were determined by qRT-PCR.

**Additional file 9: Figure S8.** Expression levels of glucose transporter genes in *M. thermophila*. **a** Relative expression levels of glucose transporter genes *MtGlt-1-1*, *MtGlt-1-2*, *MtHgt-2*, and *MtHgt-1* in glucose-rich and no-carbon conditions. Mycelia were grown in VMM supplemented with 2% sucrose for 16 h, then transferred to VMM with or without 2% glucose. After additional cultivation for 1 h, mycelia were harvested and gene expression levels were determined by qRT-PCR. **, *P* < 0.01; ***, *P* < 0.001. **b** Effect of stress on growth of WT and Δ*amyR* strains of *M. thermophila*. VMM medium with 2% (w/v) cellobiose was used. NaCl and H_2_O_2_ were added to the medium to a final concentration of 0.5 M and 1 mM, respectively. Plates were incubated at 37 °C for 4 days before imaging.

## Data Availability

All data generated or analyzed during this study are included in this published article and its additional files.
