# Supplementary figures and images for: RCO-3 and COL-26 form an external-to-internal module that regulates the dual-affinity glucose transport system in Neurospora crassa
Source: Biotechnol Biofuels. 2021 Jan 28;14:33. doi: 10.1186/s13068-021-01877-2 (PMC7841889; doi:10.1186/s13068-021-01877-2)

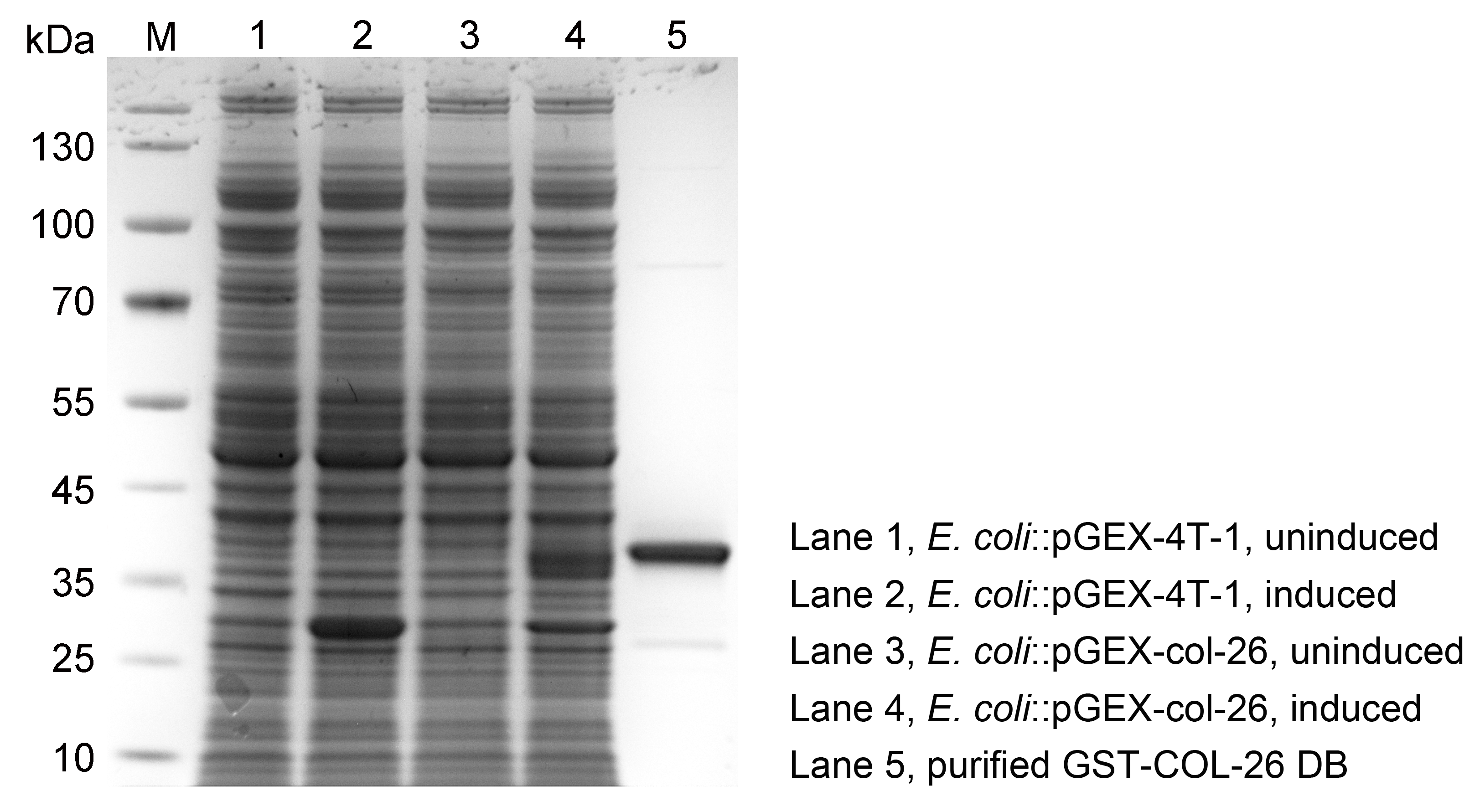

Supplement: Supplementary file 2 — Additional file 2: Figure S1. Purification of recombinant COL-26 binding domain. [file 13068_2021_1877_MOESM2_ESM.tif]

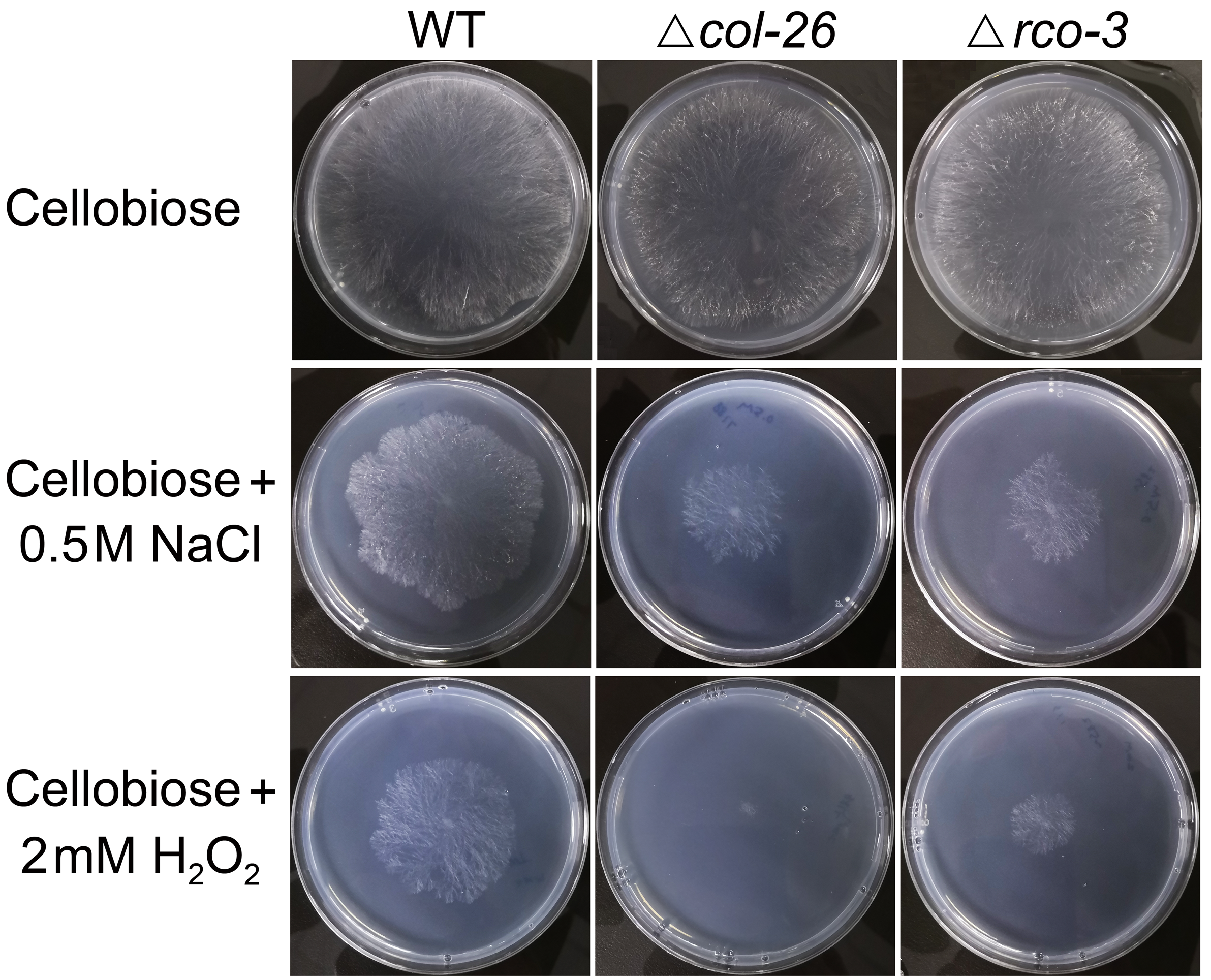

Supplement: Supplementary file 3 — Additional file 3: Figure S2. Effect of stress on the growth of WT, Δcol-26, and Δrco-3 strains of N. crassa. VMM with 2% (w/v) cellobiose was used. NaCl and H2O2 were added to the medium to a final concentration of 0.5 M and 2 mM, respectively. Plates were incubated at 28 °C in the dark for 30 h before imaging. [file 13068_2021_1877_MOESM3_ESM.tif]

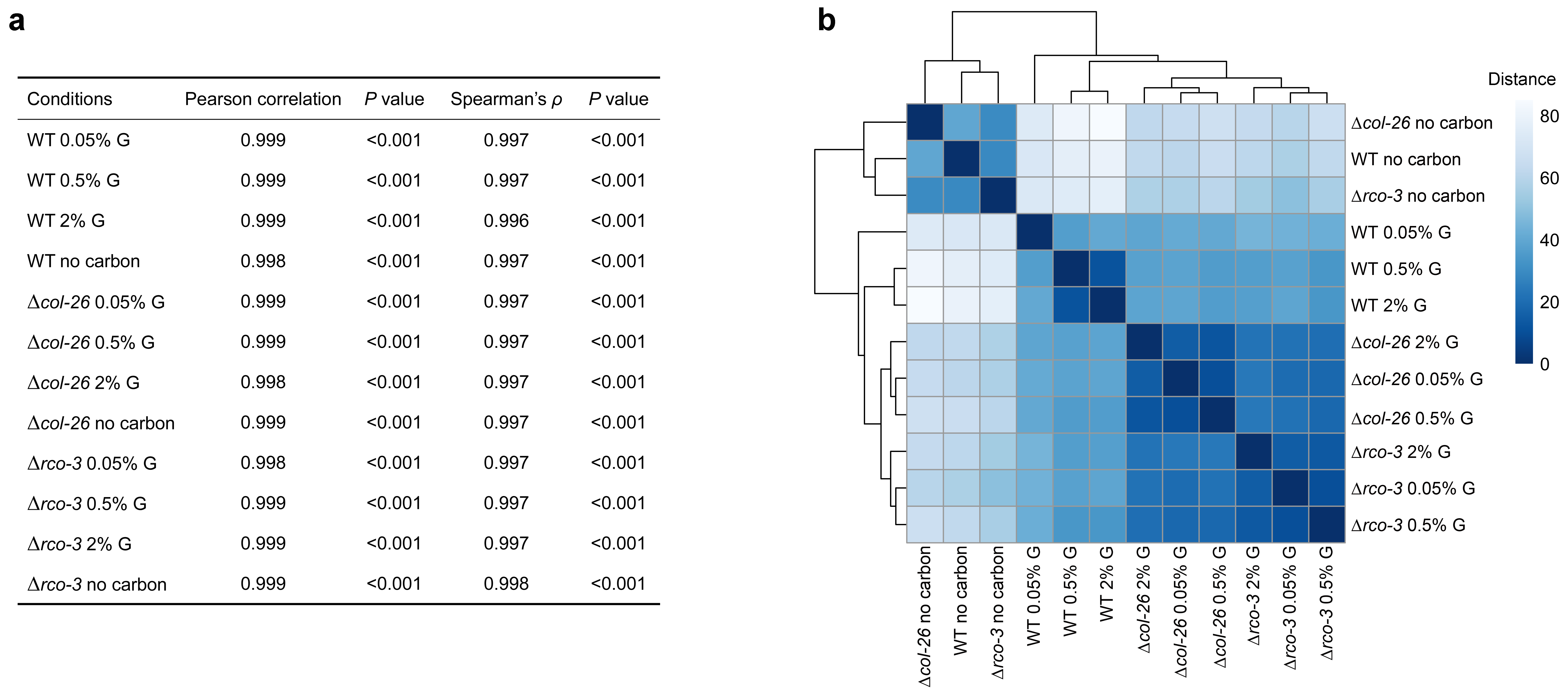

Supplement: Supplementary file 4 — Additional file 4: Figure S3. Validation of RNA-Seq data for N. crassa in response to a glucose gradient. a Spearman analysis. b Sample-to-sample clustering. [file 13068_2021_1877_MOESM4_ESM.tif]

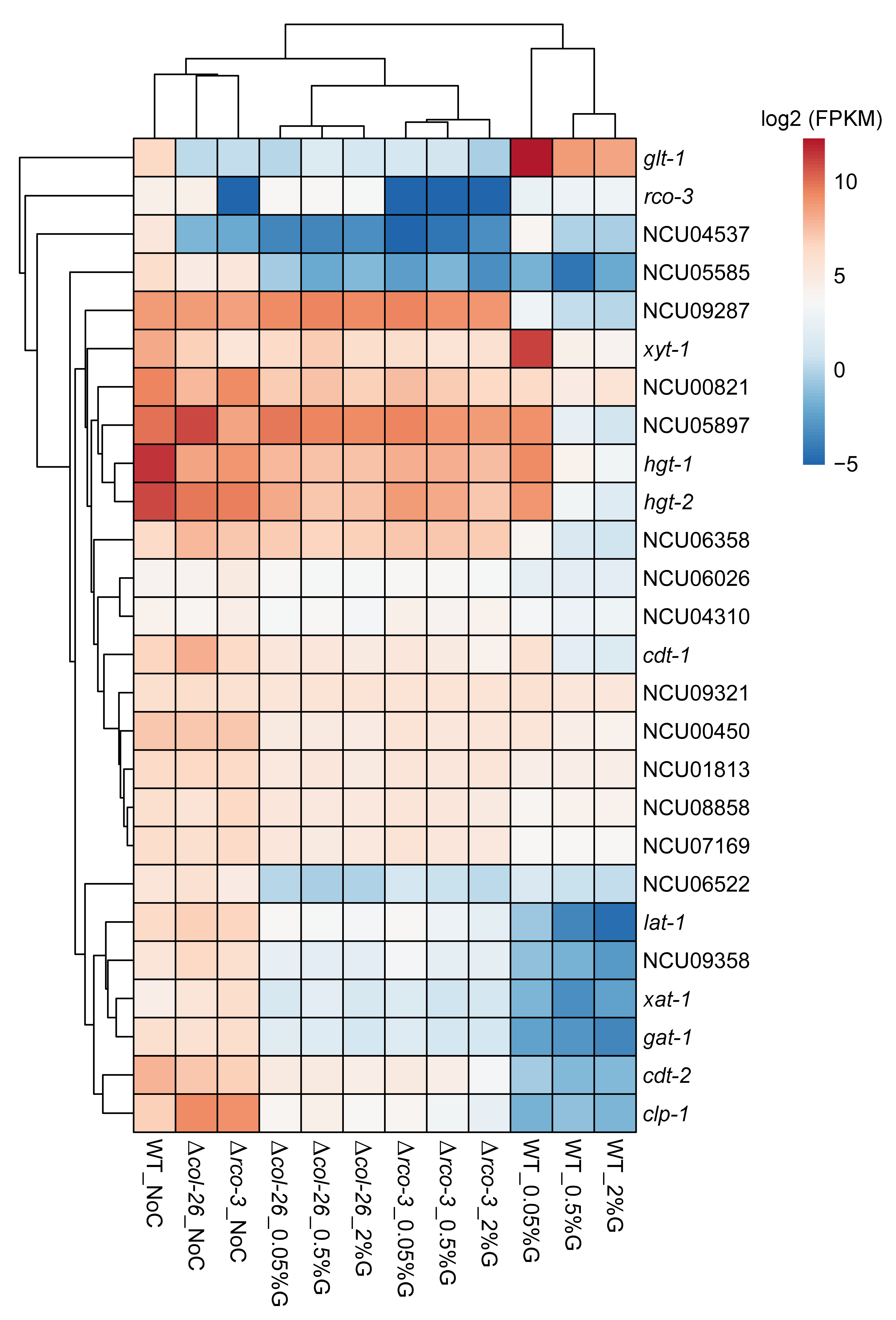

Supplement: Supplementary file 5 — Additional file 5: Figure S4. Transcriptional responses of sugar transporter genes in the WT, Δrco-3, and Δcol-26 strains of N. crassa to a glucose gradient. Heatmap analysis and clustering of 26 sugar transporter genes with robust expression levels (fragments per kilobase of transcript per million mapped reads > 20) in at least one condition. Log-transformed expression values are color-coded. [file 13068_2021_1877_MOESM5_ESM.tif]

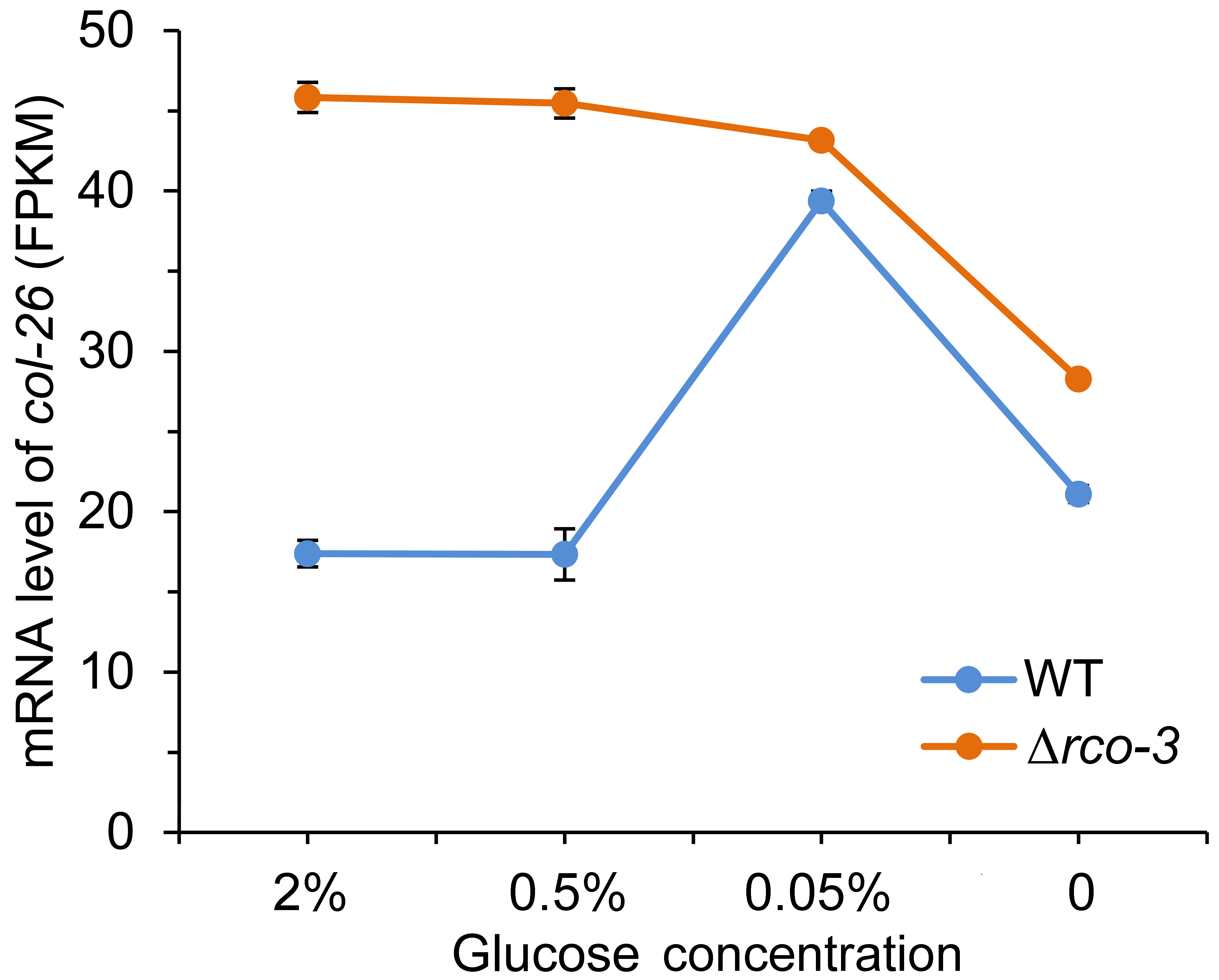

Supplement: Supplementary file 6 — Additional file 6: Figure S5. Expression levels of col-26 in WT and Δrco-3 mutant response to a glucose gradient. [file 13068_2021_1877_MOESM6_ESM.tif]

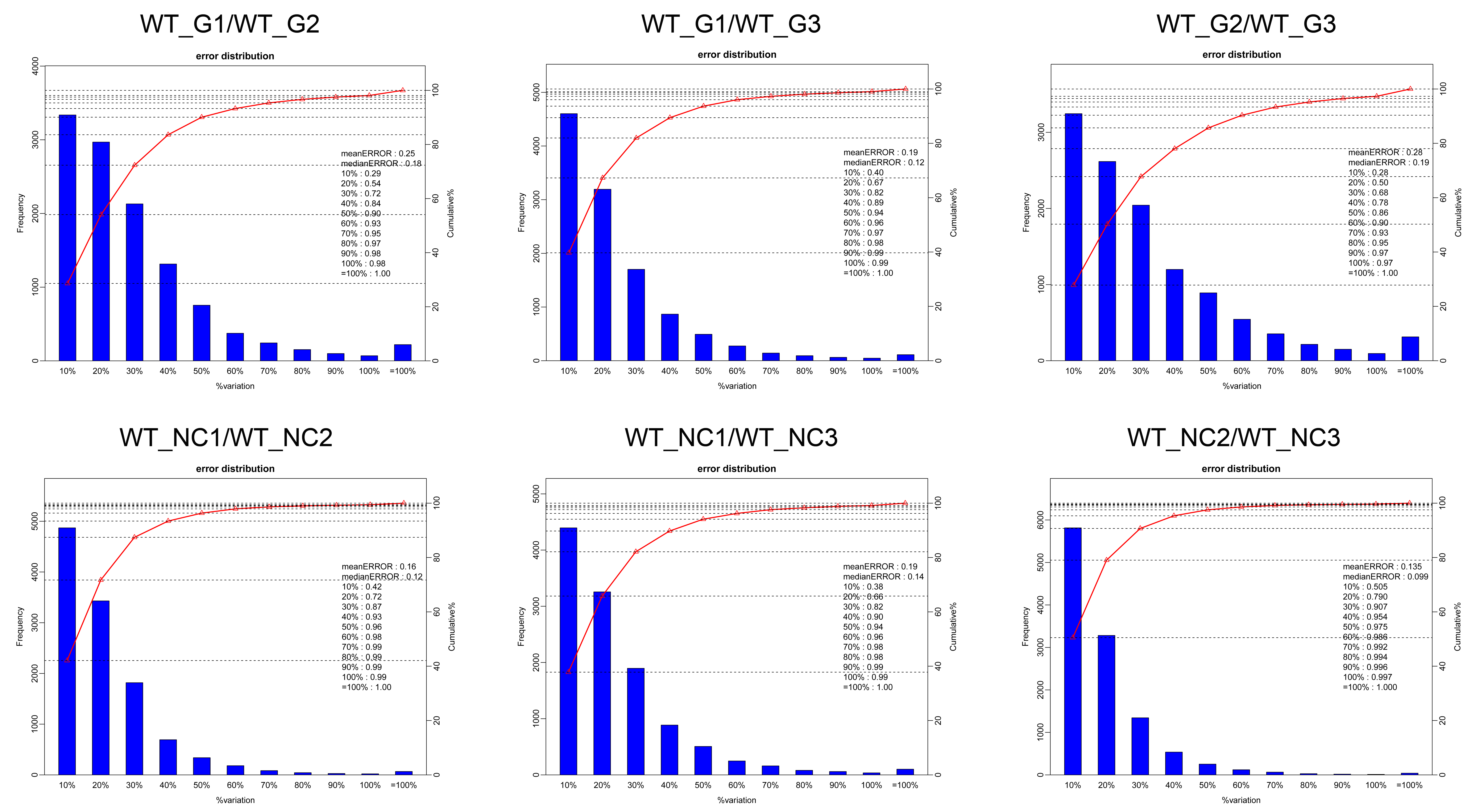

Supplement: Supplementary file 7 — Additional file 7: Figure S6. Histogram of error distribution among biological replicates of phosphoproteome in glucose-replete or no-carbon conditions. [file 13068_2021_1877_MOESM7_ESM.tif]

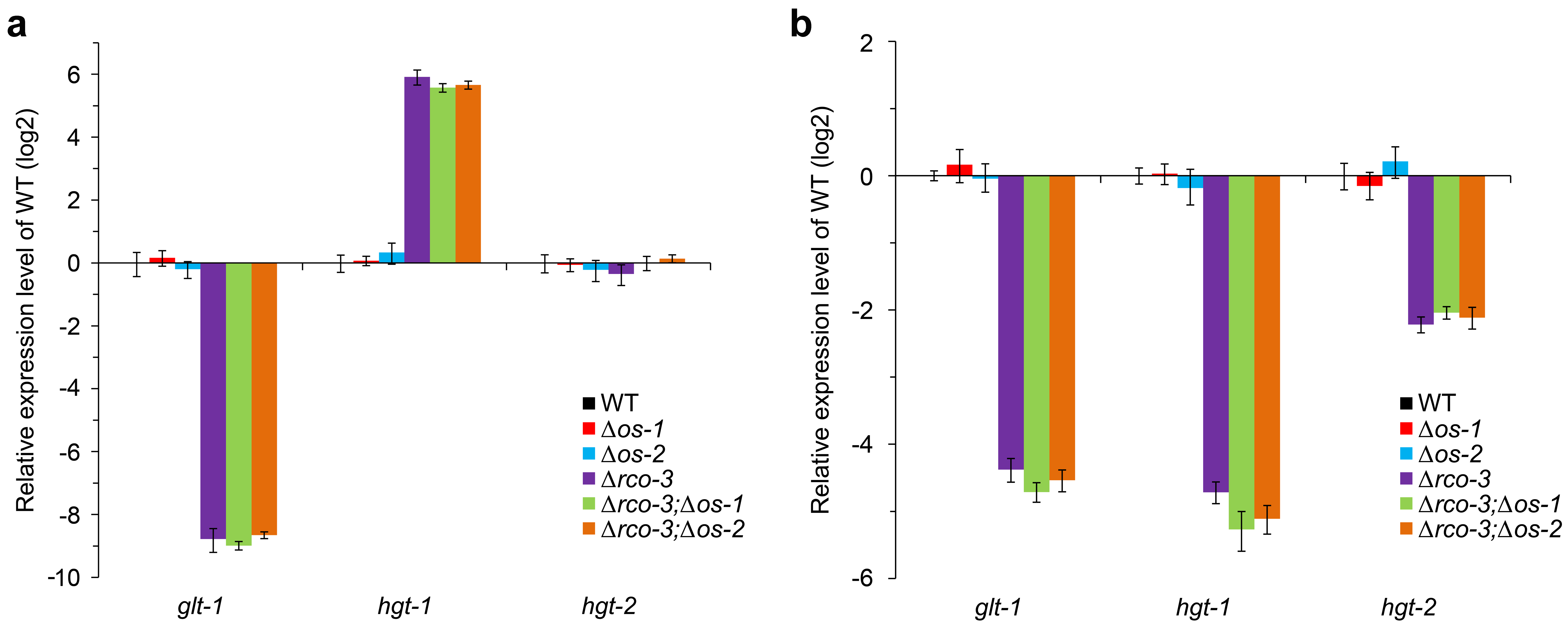

Supplement: Supplementary file 8 — Additional file 8: Figure S7. Relative expression levels of glt-1, hgt-1, and hgt-2 in WT, Δos-1, Δos-2, Δrco-3, Δrco-3;Δos-1, and Δrco-3;Δos-2 strains of N. crassa in glucose-rich (a) and no-carbon (b) conditions. Mycelia were grown in VMM supplemented with 2% sucrose for 16 h, then transferred to VMM with or without 2% glucose. After additional cultivation for 1 h, mycelia were harvested and gene expression levels were determined by qRT-PCR. [file 13068_2021_1877_MOESM8_ESM.tif]

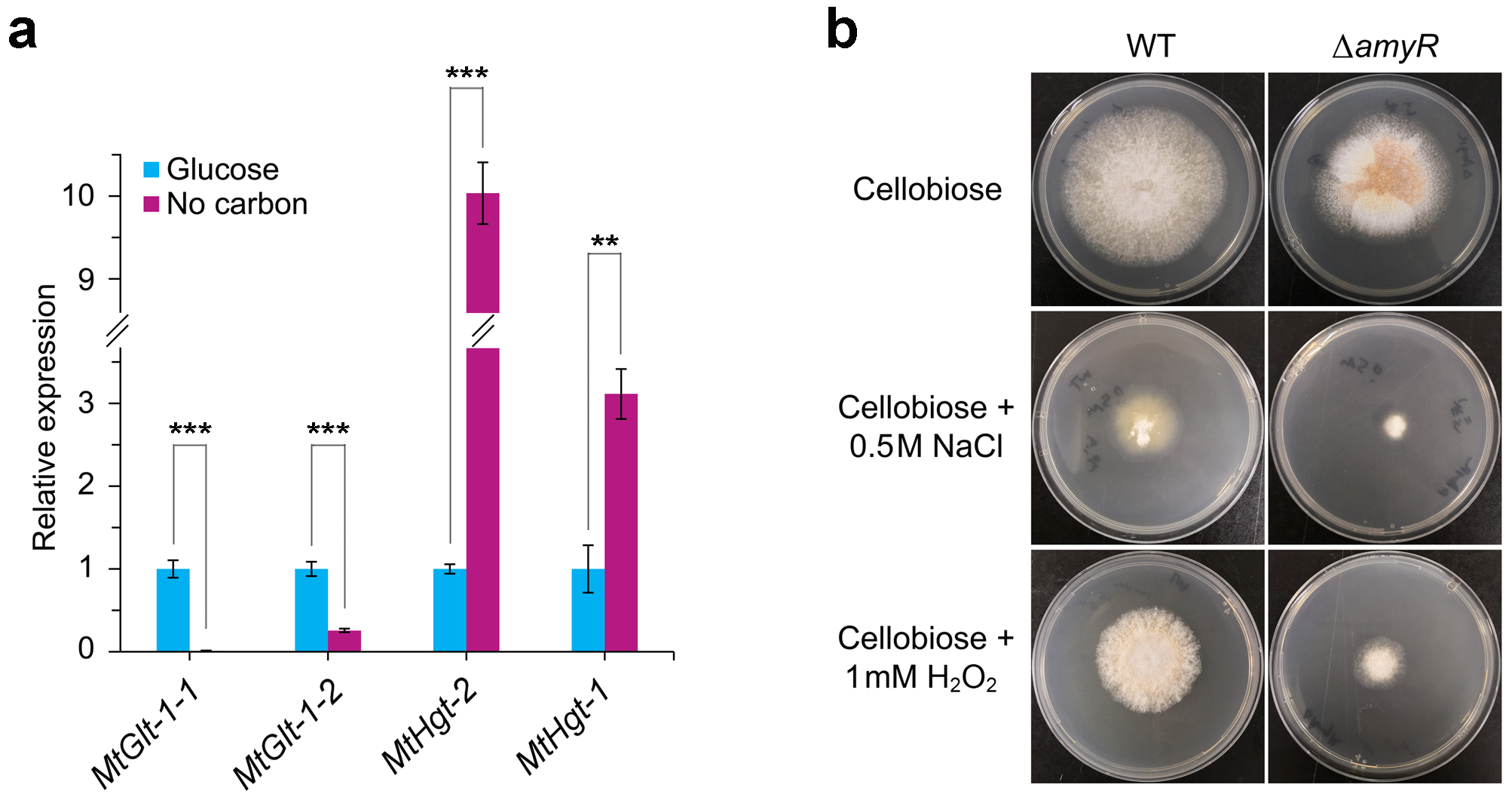

Supplement: Supplementary file 9 — Additional file 9: Figure S8. Expression levels of glucose transporter genes in M. thermophila. a Relative expression levels of glucose transporter genes MtGlt-1-1, MtGlt-1-2, MtHgt-2, and MtHgt-1 in glucose-rich and no-carbon conditions. Mycelia were grown in VMM supplemented with 2% sucrose for 16 h, then transferred to VMM with or without 2% glucose. After additional cultivation for 1 h, mycelia were harvested and gene expression levels were determined by qRT-PCR. **, P < 0.01; ***, P < 0.001. b Effect of stress on growth of WT and ΔamyR strains of M. thermophila. VMM medium with 2% (w/v) cellobiose was used. NaCl and H2O2 were added to the medium to a final concentration of 0.5 M and 1 mM, respectively. Plates were incubated at 37 °C for 4 days before imaging. [file 13068_2021_1877_MOESM9_ESM.tif]
